# The Bright Side of Curcumin: A Narrative Review of Its Therapeutic Potential in Cancer Management

**DOI:** 10.3390/cancers16142580

**Published:** 2024-07-18

**Authors:** Andrea Amaroli, Isabella Panfoli, Matteo Bozzo, Sara Ferrando, Simona Candiani, Silvia Ravera

**Affiliations:** 1 BIO-Photonics Overarching Research Laboratory (BIOPHOR), Department of Earth, Environmental and Life Sciences (DISTAV), University of Genoa, 16132 Genoa, Italy; matteo.bozzo@unige.it (M.B.); sara.ferrando@unige.it (S.F.); simona.candiani@unige.it (S.C.); 2 Department of Pharmacy (DIFAR), University of Genoa, 16132 Genoa, Italy; panfoli@difar.unige.it; 3 IRCCS Ospedale Policlinico San Martino, 16132 Genoa, Italy; 4 Department of Experimental Medicine (DIMES), University of Genoa, 16132 Genoa, Italy

**Keywords:** turmeric, curcuminoid, photochemotherapies, photodynamic therapy, drug targeting, drug delivery, nanoparticle, oxidative phosphorylation, cell metabolism, oxidative stress, tumor, anticancer agent, antitumor drugs, cancer, chemotherapy

## Abstract

**Simple Summary:**

This review comprehensively examines curcumin’s therapeutic potential in cancer treatment. It addresses the limitations of curcumin therapy due to its low bioavailability and potential side effects. It discusses how modern approaches can overcome these limitations to support its consistent and effective use in cancer therapy. Indeed, the role of curcumin in photodegradation and photodynamic therapy is emphasized through its use in combination with phototherapy. In addition, improved therapeutic efficacy, increased cellular uptake, and enhanced cytotoxicity have been demonstrated in various cancer models using curcumin-loaded nanoparticle drug delivery. Overall, the present review highlights the promising impact of curcumin in cancer treatment and the importance of optimizing its therapeutic efficacy, considering potential interactions with drugs used to manage side effects and collateral effects of cancer treatment.

**Abstract:**

Curcumin, a polyphenolic compound derived from Curcuma longa, exhibits significant therapeutic potential in cancer management. This review explores curcumin’s mechanisms of action, the challenges related to its bioavailability, and its enhancement through modern technology and approaches. Curcumin demonstrates strong antioxidant and anti-inflammatory properties, contributing to its ability to neutralize free radicals and inhibit inflammatory mediators. Its anticancer effects are mediated by inducing apoptosis, inhibiting cell proliferation, and interfering with tumor growth pathways in various colon, pancreatic, and breast cancers. However, its clinical application is limited by its poor bioavailability due to its rapid metabolism and low absorption. Novel delivery systems, such as curcumin-loaded hydrogels and nanoparticles, have shown promise in improving curcumin bioavailability and therapeutic efficacy. Additionally, photodynamic therapy has emerged as a complementary approach, where light exposure enhances curcumin’s anticancer effects by modulating molecular pathways crucial for tumor cell growth and survival. Studies highlight that combining low concentrations of curcumin with visible light irradiation significantly boosts its antitumor efficacy compared to curcumin alone. The interaction of curcumin with cytochromes or drug transporters may play a crucial role in altering the pharmacokinetics of conventional medications, which necessitates careful consideration in clinical settings. Future research should focus on optimizing delivery mechanisms and understanding curcumin’s pharmacokinetics to fully harness its therapeutic potential in cancer treatment.

## 1. Introduction

**Figure 1:**
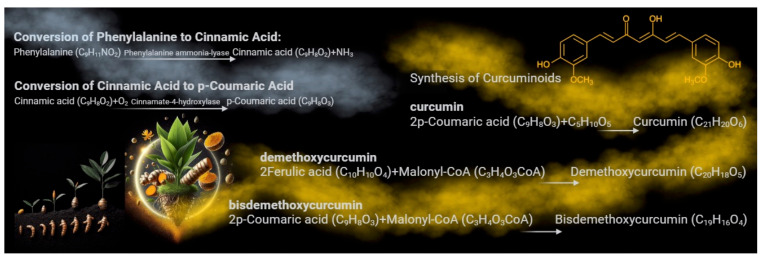
The biosynthesis pathway of curcumin in turmeric. The biosynthesis of curcumin in the rhizomes of Curcuma longa involves several enzymatic steps. It begins with phenylalanine ammonia-lyase converting phenylalanine into cinnamic acid. Cinnamic acid is then converted into p-coumaric acid, a key intermediate. Through a series of enzymatic reactions, p-coumaric acid forms curcuminoid precursors such as curcumin, demethoxycurcumin, and bis-demethoxycurcumin. Notably, demethoxycurcumin is formed from the intermediate ferulic acid derived from p-coumaric acid through methylation and other modifications. These rhizome processes contribute to the plant’s defense mechanisms and protection against oxidative stress and pathogens.

Curcumin, a polyphenolic compound, is synthesized in the rhizome of *Curcuma longa*, or turmeric [1,2]. This process involves several enzymatic steps within the plant. It starts with phenylalanine ammonia-lyase converting phenylalanine into cinnamic acid, a critical step in the biosynthesis of phenylpropanoids, of which curcumin is a part [3]. Cinnamic acid then undergoes a series of enzymatic reactions in which p-coumaric acid acts as a key intermediate compound, forming curcumin precursors known as curcuminoids [3]. These precursors, including curcumin, demethoxycurcumin, and bis-demethoxycurcumin, are further modified through a series of enzymatic reactions [4]. These reactions lead to the formation of molecules commonly known as curcumin, the main active compound responsible for turmeric’s therapeutic activity and characteristic yellow-orange color [5] (Figure 1). This biosynthesis and accumulation process occurs in the rhizomes of the turmeric plant, where it serves various functions, including protection against oxidative stress and defense against pathogens [1].

Curcumin, found in approximately 130 species of Curcuma, has a long history of traditional medicinal use [6]. Mainly, *Curcuma longa* (turmeric), *Curcuma aromatica* (wild turmeric), and *Curcuma xanthorrhiza* (Javanese turmeric) have been used for health maintenance and disease management, dating back to ancient Indian and Chinese medicine over 4000 years ago [6,7,8]. In these traditional practices, turmeric, containing curcumin, was used to treat various conditions such as respiratory disorders, liver diseases, anorexia, rheumatism, and diabetic wounds [9]. Despite the long history of traditional medicinal use, a comprehensive understanding of the therapeutic actions and health benefits remains to be validated.

Curcumin, as highlighted in the literature, holds immense promise in health. Its antioxidant properties enable it to neutralize free radicals and protect cells from oxidative damage, while its anti-inflammatory action inhibits the expression of inflammatory mediators and reduces inflammation at the cellular level [10]. Curcumin has demonstrated neuroprotective effects and potential benefits in treating neurodegenerative diseases. Recent studies have underscored the role of curcumin in reducing brain inflammation, protecting neurons, and promoting neurogenesis [1]. Moreover, numerous studies have illuminated the anticarcinogenic potential of curcumin in various types of cancer, including colon, pancreatic, and other high-risk cancers. Curcumin has been studied for its ability to inhibit tumor cell growth, induce apoptosis, and reduce resistance to chemotherapy and radiotherapy [1,11].

**Figure 2:**
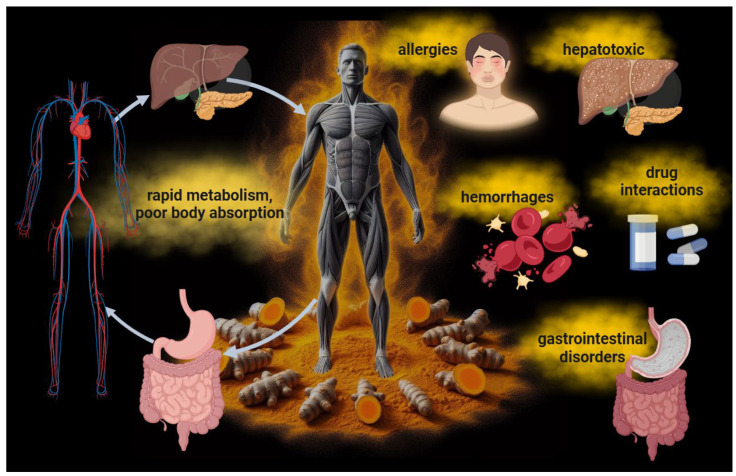
The limitations in the therapeutic application of curcumin in patients. Curcumin’s low bioavailability due to its poor water solubility, rapid hepatic metabolism, and low intestinal absorption results in ineffective absorption by the body. The necessity of using high concentrations of the molecule can lead to side effects such as allergies, gastrointestinal disturbances, hepatotoxicity, and sometimes anticoagulant and antiplatelet effects. Curcumin may also interact with certain medications, including anti-inflammatory, cardiovascular, antibiotic, and antitumor drugs.

While the positive effects of curcumin have been observed mainly in in vitro experiments, it is crucial to be aware of its in vivo limitations (Figure 2). Once administered, its low absorption and rapid metabolism might compromise its bioavailability and clinical applicability. Curcumin has poor water solubility, rapid hepatic metabolism, and low intestinal absorption [12,13]. This means that the body may not effectively absorb a large amount of ingested curcumin. In particular, after ingestion, the liver rapidly metabolizes curcumin into compounds with lower biological activity than curcumin itself, limiting the amount of the active molecule that reaches the systemic circulation and potential targets [12]. This necessitates the intake of high concentrations of curcumin to achieve efficient bioavailability and therapeutic activity. Curcumin is generally considered safe and well tolerated, with the FDA classifying it as “generally recognized as safe”. However, high doses of curcumin can cause tolerance issues that may be unmanageable for patients, such as experiencing allergies, gastrointestinal disturbances such as nausea, diarrhea, or stomach discomfort, as well as issues related to its hepatotoxicity [14,15]. Moreover, curcumin might affect blood coagulation. Numerous studies indicate that curcumin possesses anticoagulant and antiplatelet properties. Specifically, it has been shown that curcumin can prolong activated partial thromboplastin time and prothrombin time, both tests measuring blood coagulation levels. These effects suggest that curcumin can slow blood clotting, reducing the risk of clot formation [16]. Additionally, curcumin has been shown to inhibit platelet activation and aggregation, further contributing to its antithrombotic properties. This effect could be particularly useful in preventing thrombosis and atherothrombosis, which are significant risk factors for cardiovascular diseases [17]. However, it is essential to note that although curcumin may offer benefits as a natural anticoagulant, its use should be carefully monitored, especially in combination with other anticoagulant drugs, as it could amplify the effect of these drugs and increase the risk of excessive bleeding. Therefore, anyone already undergoing anticoagulant treatment should consult their doctor before taking curcumin supplements or turmeric-based products. Moreover, curcumin may also interact with some medications, such as anti-inflammatory and antitumor drugs [18].

## 2. Well-Known Curcumin Effects on Cancer Cell Growth and Proliferation

### 2.1. Inhibition of Cell Proliferation and Induction of Apoptosis

**Figure 3:**
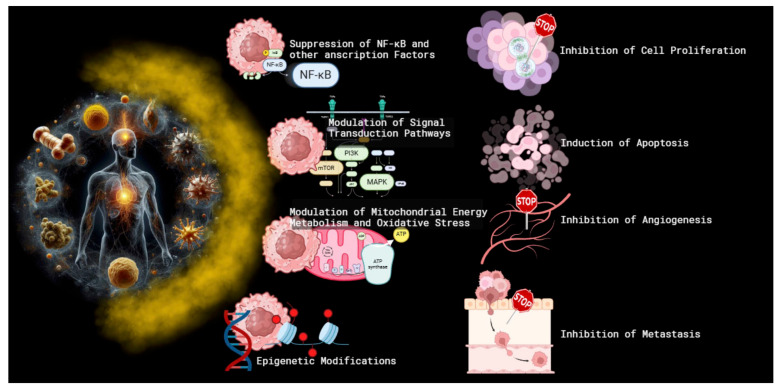
The mechanisms of curcumin’s anticancer effects. Curcumin, a polyphenolic compound derived from turmeric, exerts its anticancer effects through multiple mechanisms. These include the inhibition of cell proliferation and the induction of apoptosis via cell cycle arrest and the modulation of apoptotic proteins. Curcumin suppresses the activity of key transcription factors like NF-κB, STAT3, and AP-1 and interferes with critical signal transduction pathways such as PI3K/Akt/mTOR and MAPK/ERK. Additionally, curcumin inhibits angiogenesis and metastasis by downregulating VEGF, VEGFR2, and matrix metalloproteinases (MMPs). Epigenetic modifications through the inhibition of DNA methyltransferases (DNMTs) and histone deacetylases (HDACs) further contribute to its anticancer properties. Finally, curcumin alters mitochondrial energy metabolism and reduces oxidative stress by inhibiting FoF1-ATP synthase, thereby impacting ATP production and reactive oxygen species (ROS) generation, which are crucial for cancer cell growth and proliferation.

One of the primary mechanisms by which curcumin exerts its anticancer effects is through the inhibition of cell proliferation and the induction of apoptosis [19,20,21]. Studies have demonstrated that curcumin can suppress the proliferation of cancer cells by interfering with the cell cycle [21,22]. Specifically, it induces cell cycle arrest at the G2/M phase in several cancer types [23,24,25,26], modulating the level of cyclin-dependent kinases (CDKs) and cyclins through the increased expression of CDK inhibitors [27,28]. Furthermore, curcumin induces apoptosis through both intrinsic and extrinsic pathways [29,30,31]. Curcumin enhances the expression of pro-apoptotic proteins such as Bax, Bak, PUMA, Bim, and Noxa and death receptors such as TRAIL-R1/DR4 and TRAIL-R2/DR5 [31,32,33,34]. In addition, curcumin decreases the levels of anti-apoptotic proteins like Bcl-2, Bcl-XL, survin, and XIAP [32,35]. This shift in the balance of apoptotic regulators facilitates the release of cytochrome c from mitochondria [33,35] and activates caspases [35,36,37,38], leading to programmed cell death (Figure 3).

### 2.2. Suppression of NF-κB and Other Transcription Factors

Curcumin’s anticancer efficacy is also attributed to its ability to inhibit nuclear factor kappa B (NF-κB) [39,40,41,42,43], a transcription factor that plays a pivotal role in cancer cell survival, proliferation, and metastasis [44]. NF-κB is often constitutively active in various cancers, promoting the expression of genes involved in inflammation, cell survival, and angiogenesis [45]. Curcumin suppresses NF-κB activation by inhibiting IκB kinase (IKK), thereby preventing the phosphorylation and degradation of IκBα, an inhibitor of NF-κB [46,47,48], and reducing the transcription of NF-κB target. In addition to NF-κB, curcumin also modulates other transcription factors such as STAT3 and AP-1 [49,50,51,52,53]. By inhibiting these factors, curcumin reduces the expression of genes involved in cell proliferation and survival, contributing to its anticancer properties [49,50,51,52,53].

### 2.3. Modulation of Signal Transduction Pathways

Curcumin’s impact on various signal transduction pathways further elucidates its multifaceted anticancer effects. It has been shown to interfere with the PI3K/Akt/mTOR pathway [52,54], a critical signaling axis for cell growth and survival. Curcumin inhibits the phosphorylation of Akt [54,55], leading to the suppression of downstream targets involved in cell proliferation and survival. Additionally, curcumin affects the MAPK/ERK pathway [53,56], which is implicated in cell differentiation, proliferation, and apoptosis. By inhibiting this pathway, curcumin can reduce cancer cell growth and induce apoptosis.

### 2.4. Inhibition of Angiogenesis and Metastasis

Curcumin also exerts anti-angiogenic and anti-metastatic effects [57,58,59], which are crucial for limiting tumor growth and spread. Curcumin inhibits angiogenesis by downregulating the expression of vascular endothelial growth factor (VEGF) and its receptor VEGFR2 [60,61]. This inhibition prevents the proliferation and migration of endothelial cells, thereby reducing blood vessel formation. Moreover, curcumin inhibits metastasis by modulating the expression of matrix metalloproteinases (MMPs) [43,62,63], enzymes involved in the degradation of the extracellular matrix, a key step in cancer cell invasion and metastasis. Curcumin downregulates MMP-2 and MMP-9 [63], thereby impairing the invasive capabilities of cancer cells. Also, it inhibits the chemokine CXCL12/CXCR4 axis, whose activation is involved in tumor epithelial–mesenchymal transition (EMT), cancer cell motility, and metastasis [64].

### 2.5. Epigenetic Modifications

Emerging evidence suggests that curcumin can also exert its anticancer effects through epigenetic modifications [65]. These modifications include the inhibition of DNA methyltransferases (DNMTs) and histone deacetylases (HDACs), which play a role in gene expression regulation [66]. By modulating these epigenetic factors, curcumin can reactivate tumor suppressor genes and inhibit oncogenes [67,68], contributing to its anticancer activity.

### 2.6. Alteration in Mitochondrial Energy Metabolism and Related Oxidative Stress Production

Although the energy metabolism of cancer cells appears to be supported more by anaerobic glycolysis (the Warburg effect), mitochondria play a pivotal role in cancer cell physiology, driving both energy production and the biosynthetic processes essential for rapid proliferation [69]. Several studies reported that cancer cells often exhibit altered mitochondrial function, characterized by enhanced oxidative phosphorylation (OXPHOS) and increased mitochondrial biogenesis [70,71]. These adaptations support the high metabolic demands of tumorigenesis [72], since mitochondrial function reprogramming can confer resistance to chemotherapy and contribute to metastatic potential [73,74]. Beyond energy production, mitochondria play a pivotal role in cancer cell growth and proliferation, primarily through the generation of reactive oxygen species (ROS) [75,76,77]. A mild ROS concentration induces the activation of signaling pathways such as MAPK, PI3K/Akt, and NF-κB, which are involved in cell proliferation and survival [78,79]. Moreover, oxidative stress can induce the expression of growth factors and cytokines, enhancing tumor progression and metastasis [79]. In this scenario, curcumin appears to be one of the most promising molecules to modulate OxPhos activity and the related oxidative stress production [80]. The literature reports that curcumin directly inhibits the activity of F_o_F_1_ -ATP synthase (ATP synthase) [39,81], as it binds to the F1 moiety through its 4′hydroxy groups and a β-diketone [82], reducing the available energy to support the proliferation and growth of cancer cells. In addition, ATP synthase inhibition not only impacts ATP production but also modulates oxidative damage. In coupling conditions, ATP synthase reduction slows down electron transport chain (ETC) function and the relative ROS production [81], switching off the proliferation signaling associated with the pro-oxidant environment. By contrast, when mitochondria are damaged, curcumin concurs with the oxidative stress increment [83].

## 3. Improving Curcumin’s Therapeutic Effectiveness: How Light Affects Curcuminoids—Photodegradation vs. Photodynamic Therapy

### 3.1. Photo-Oxidation and Photoisomerization

The commercial form of turmeric root, commonly known as “curcumin”, is a mixture of three compounds: monomolecular curcumin or pure curcumin, demethoxycurcumin, and bis-demethoxycurcumin at a ratio of about 80:15:5. Therefore, molecules such as demethoxycurcumin and bis-demethoxycurcumin are available in smaller amounts than monomolecular curcumin [84]. However, these molecules significantly affect curcumin’s stability and its biological interactions. Studies indicated that demethoxycurcumin and bis-demethoxycurcumin affect monomolecular curcumin’s stability under certain light conditions [85]. The photophysical properties of curcumin are closely related to its chemical nature. It is a polyphenol with a diarylheptanoid structure consisting of two aromatic rings linked by a chain of seven carbon atoms. Its molecular formula is 1,7-bis(4-hydroxy-3-methoxyphenyl)hepta-1,6-diene-3,5-dione (C21H20O6). C20H18O6 is the formula for demethoxycurcumin and C19H16O6 is for bis-demethoxycurcumin [86,87]. Furthermore, curcumin’s molecular structure exhibits keto–enol tautomerism, with different conformations depending on the solvent and pH. The keto form is predominant under acidic and neutral conditions, whereas the enol form is present under alkaline conditions [88]. It is important to note that the enol form of curcumin is the most stable in an organic solution. This is because it exhibits greater electron delocalization within the conjugated bond system. It absorbs light in the visible region, with a maximum absorption of around 420 nm. The keto form is less stable and tends to convert rapidly to enol. The keto form absorbs light at higher wavelengths than the enol form, with a maximum absorption of around 500 nm, due to the position of the double bond within the molecule [89]. Furthermore, Bernd [90] highlighted that considering the cis or trans conformation of curcumin is important when studying its interaction with light. Curcumin can exist in two isomeric forms that can absorb light in different regions of the electromagnetic spectrum. The cis form has absorption peaks at wavelengths around 405 nm, whereas the trans is known to have absorption peaks around 420 nm. This difference in light absorption between the two isomeric forms can influence how they interact with visible and UV light at low doses, potentially affecting their therapeutic properties. The modification of curcumin under light exposure can influence its bioactivity profile due to the initiation of photochemical reactions (photodegradation, including photo-oxidation, photoisomerization, and photoactivation/photosensitization). Light can lead to changes in its molecular structure [85]. The breaking of chemical bonds, degradation, and the formation of new molecules can occur after the exposure of curcumin to visible light and UV radiation. These events may affect the stability of curcumin and its ability to maintain its desired biological properties [90]. The photodegradation induced by UV light in the solid state and solution can lead to the fragmentation of the dicarbonyl system, as Tønnesen et al. [91] reported. In particular, the process of photo-oxidation of curcumin is a complex phenomenon. The absorption of light energy at specific wavelengths, such as UV or visible light, triggers curcumin, placing it in an excited and reactive state [92]. This excited state can lead to oxidation reactions within the curcumin molecule, forming degradation products. The photo-oxidation products of curcumin can vary depending on the experimental conditions, such as the type of light used and the presence of solvents or other reactive compounds. The photo-oxidation of curcumin can remove a methoxy group (-OCH3) to form demethoxycurcumin. Subsequently, demethoxycurcumin can undergo further oxidation, resulting in the loss of another methoxy group to form bis-demethoxycurcumin [88,92,93,94]. Considering the more general degeneration process of curcumin degradation, shorter phenols such as vanillin, ferulic acid, vanillic acid, and ferulaldehyde are formed, as well as more complex and not yet fully characterized mixtures [85,95]. Recently, Ravera et al. [96] demonstrated how irradiation with a 450 nm wavelength diode laser with parameters of 0.25 W, 15 J, 60 s, and 1 cm^2^ and in continuous wave mode was capable of inducing curcumin in a DMSO solution to generate degradation products exhibiting absorption peaks at 280 nm and 350 nm, in addition to the classical peak around 420 nm. Compared to monomolecular curcumin, the degradation products enhanced antitumor activity toward a head and neck squamous cell carcinoma cell line. Chatterjee and colleagues [93] have shown how UV-A light can induce changes in the molecular configuration of curcumin using techniques such as drift-tube ion mobility mass spectrometry, high-performance liquid chromatography, and collision-induced dissociation of selected molecular ions. In particular, changes around the C=C bonds led to two curcumin photoisomers forming precursors for forming isomeric dimers via a [2 + 2] cycloaddition reaction and H_2_ loss products. Marazzi and colleagues [94] identified possible trans-to-cis photoisomerization of cyclocurcumin upon exposure to visible wavelengths ranging from about 400 nm (blue-violet) to 700 nm (red). In many cases, the photo-derived products retain a conjugated structure. They can exhibit photophysical characteristics similar to those of curcumin, allowing them to interact with light and potentially play biological or chemical roles. However, their properties may differ slightly from the original curcumin [88].

### 3.2. Photodynamic Therapy

**Figure 4:**
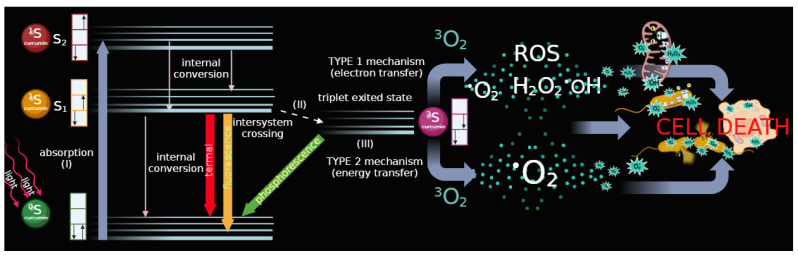
Photodynamic therapy mechanism of action. Upon irradiation, the photosensitizer (e.g., curcumin) transitions from the ^0^S ground state to the ^1^S excited singlet state and then, via intersystem crossing, to the ^3^S triplet. The longer-lived triplet interacts with surrounding molecules, generating cytotoxic species such as reactive oxygen species (ROS). These include singlet oxygen (-O^2^), a hydroxyl radical (-OH), and hydrogen peroxide (H_2_O_2_). PDT is divided into two types based on the tissue’s oxygen concentration: both involve the transition from the basic singlet state (^0^S) to the excited singlet state (^1^S). In the Type I mechanism, reactions with the excited sensitizer produce free radicals and reactive oxygen species, which cause oxidative damage. In the Type II mechanism, the excited triplet transfers energy to molecular oxygen, producing singlet oxygen. This interacts with biological substrates, leading to oxidative damage and cell death.

The photoactivation/photosensitization process of curcumin molecules is responsible for supporting photodynamic therapy (PDT) [97,98,99]. In summary, when exposed to light, the photosensitizer (such as curcumin) is excited from its ground state to the first excited singlet state and then transitions to the triplet state via intersystem crossing. The longer lifespan of the triplet state allows the excited photosensitizer to interact with surrounding molecules. It is widely accepted that this interaction leads to the production of cytotoxic species during the PDT effect. Researchers categorize PDT into two types based on the oxygen levels in the examined tissues. In both types, photosensitizers transition from the singlet ground state (0S) to the singlet excited state (1S) when exposed to light. The process of energy generation is divided into two phases: diagnostic and therapeutic. Both mechanisms lead to a transition from the singlet excited state (1S) to the triplet excited state (3S) [98,100]. The subsequent steps of the first and second phototherapy mechanisms differ significantly. The Type I mechanism involves hydrogen atom abstraction or electron transfer reactions between the excited sensitizer and a substrate, which can be a solvent or another sensitizer, resulting in the formation of free radicals and radical ions. These highly reactive radical species can interact with molecular oxygen to produce reactive oxygen species such as superoxide anions or hydroxyl radicals, causing oxidative damage and biological lesions. In contrast, the Type II mechanism involves energy transfer between the excited triplet state of the sensitizer and ground-state molecular oxygen, producing singlet oxygen, the first excited state of oxygen. These zwitterionic species are highly reactive and can interact with various biological substrates, leading to oxidative damage and cell death. Type II processes are generally considered dominant in PDT, with singlet oxygen being the main cytotoxic agent responsible for the biological effects [101]. Type I reactions, however, become more significant at low oxygen concentrations or in more polar environments [102]. After irradiating the photosensitizer with light of a specific wavelength, biochemical and physiological processes occur, leading to intermediate and final effects at the cellular level [97] (Figure 4). Reactive oxygen species (ROS) are generated, in particular, singlet oxygen, hydroxyl radicals, and hydrogen peroxide. The occurrence of damage to lipids, proteins, and nucleic acids is observed. This damage leads to mitochondrial dysfunction and cellular imbalance, resulting in cellular stress signaling, such as increased nuclear factor kappa-light-chain-enhancer of activated B cells (NF-kB) transcription factor and increased production of inflammatory cytokines. Subsequent cell death occurs directly through apoptosis and necrosis. It can also occur indirectly by activating the immune response. [97,98,103].

#### 3.2.1. Curcumin-Mediated Photodynamic Therapy: Impact on Potential Tumorigenic Microorganisms

Curcumin PDT has been utilized and investigated across various cancer cell types and a spectrum of microorganisms and viruses [104,105]. It is important to note that curcumin itself can impede bacterial growth and thwart virulence factors through multiple mechanisms, including the inhibition of the bacterial quorum sensing system, pivotal for biofilm formation and virulence factor expression; the suppression of extracellular polymeric substances (EPSs), crucial components of biofilms facilitating bacterial growth; the targeting of cellular structures such as cell membranes, cell walls, proteins, and DNA, resulting in bacteriostatic and bactericidal effects while restraining the expression of virulence factors [106,107,108,109,110,111]. However, activating curcumin via PDT with blue light can enhance its capacity to induce toxicity and inhibit bacterial growth. This process may refine curcumin targeting and reduce the required dosage and exposure times to achieve an antibacterial effect [106].

Pilegi et al. [112] investigated the effectiveness of curcumin-mediated PDT inactivation against Enterococcus faecalis. They discovered that a concentration of 5 μM of curcumin, coupled with light irradiation at 450 mW/cm^2^ for 4 min and a pre-illumination time of 30 min, resulted in a viability reduction of 7 logs in planktonic cultures compared to the negative control group.

Curcumin demonstrated its broad spectrum of antimicrobial activity on Streptococcus mutans biofilms, with a concentration of 40 μM, an LED with an intensity of 19 mW/cm^2^, and a wavelength of 450 nm, significantly decreasing biofilm viability. Similarly, concentrations of 5, 10, 20, 30, and 40 μM of curcumin and light irradiation doses of 5.28 J/cm^2^ and 18 J/cm^2^ affected Candida albicans biofilms [113].

The antimicrobial effect of curcumin photodynamic therapy on Porphyromonas gingivalis and Aggregatibacter actinomycetemcomitans was achieved under specific conditions. This included irradiating with 20 and 40 μmol/L concentrations and using a 450–470 nm LED wavelength and a 300 mW/cm^2^ power density [114].

Photodynamic therapy with a 450 nm laser light, a fluence of 10 J/cm^2^, and a curcumin concentration of 500 μg/mL significantly impacted the viability of Leishmania major and Leishmania braziliensis; even 31.25 μg/mL showed some effect [115].

Despite not representing a direct treatment of tumor cells, the possibility of utilizing the effects of curcumin PDT on bacterial consortia appears of great interest in the prevention of various forms of cancer. Based on research observations and comparisons, more authors propose that cancer cells may have a bacterial origin, suggesting a novel perspective on the fundamental problem of cancer cell origin [116]. Moreover, the role of the microbiota in carcinogenesis has recently emerged [117]. It is now widely demonstrated that various bacteria, such as *Streptococcus mutans*, *Helicobacter pylori*, *Salmonella typhi*, *Chlamydia pneumoniae*, *Aggregatibacter actinomycetemcomitans,* and others, are associated with different types of cancers, highlighting the role of bacteria in cancer development [118]. *Monas gingivalis* and *Fusobacterium nucleatum* can act in various ways to promote carcinogenesis. *Porphyromonas gingivalis* can interact with gingival epithelial cells and accelerate cell progression through the cell cycle, the induction of pro-Matrix Metalloproteinase-9 and its activation, and the upregulation of B7 Homolog 1 (also known as PD-L1, Programmed Death-Ligand 1) and B7 Dendritic Cell (also known as PD-L2, Programmed Death-Ligand 2) receptors on oral squamous carcinoma cells [119]. Fusobacterium nucleatum increases collagenase production and epithelial cell migration, promotes colon–rectal carcinogenesis by modulating E-cadherin/beta-catenin signaling via Fusobacterium adhesin A, recruits tumor-infiltrating immune cells, and generates a pro-inflammatory microenvironment conducive to colorectal cancer progression [119]. Microorganisms can influence the tumor microenvironment, promote chronic inflammation, and interact with host cells to promote tumor growth and progression [120].

#### 3.2.2. Curcumin-Mediated Photodynamic Therapy: Impact on Tumors

Curcumin has emerged as a beacon of hope when used in photodynamic therapy for tumor treatment [49,104,121]. Studies conducted in vitro and on animal models have illuminated curcumin’s potential to modulate various molecular responses, inhibiting inflammatory and pro-survival pathways related to transcription factors like NF-κB or Activator Protein 1 [122]. Curcumin increases the likelihood of apoptosis in tumor cells and stimulates the production of radicals capable of eliminating such cells through the PDT-activated pathway. However, curcumin’s poor water solubility, limited bioavailability, and rapid metabolism into derivatives present significant hurdles for its clinical application in cancer treatment. As a result, PDT with curcumin is accompanied by the development of stable drug carrier formulations that enhance cutaneous penetration and therapeutic efficacy while simultaneously reducing side effects.

In the realm of breast cancer treatment, PDT with curcumin has demonstrated remarkable efficacy in vitro. Machado et al. [123], using the human mammary cell line MCF-7 and curcumin concentrations of 20 μM, 40 μM, 80 μM, 100 μM, and 120 μM, performed PDT through irradiation with an LED device operating at 447 (±10) nm, with a power of 420 mW and a total power of 2.52 W, for an irradiance of 209 W/cm^2^ and a fluence of 80 J/cm^2^, and set at 6.4 s/application. The study reported a high phototoxic effect on MCF-7 cells, decreasing to less than 10% of viable tumor cells after two irradiations. This combination also increased the production of ROS and levels of caspase 3/7 activity, indicating the induction of cell apoptosis and ultimately leading to cell death. The minimum effective dose of curcumin in the experiment was 20 μM. No significant activity of caspases 3 and 7 was observed in healthy fibroblast cells of the human embryonic fibroblast cell line following treatment. The authors developed a curcumin nanoemulsion capable of stabilizing curcumin and increasing its solubility to enhance curcumin’s bioavailability.

Sun et al. [124] studied the murine breast cancer cell line 4T1. A total of 6.25 μg/mL of free curcumin or curcumin nanodrugs and irradiation at 450 nm and a power of 640 mW for 1 min induced an increased percentage of apoptotic cells. Specifically, treatment with curcumin nanodrugs led to a higher percentage of apoptosis through the c-Jun N-terminal Kinase/caspase-3 mediated pathway compared to free curcumin.

Khorsandi et al. [125], using the human breast cancer cell line MDA-MB-231, curcumin concentrations of 25 and 100 μg/mL, and irradiation with a blue LED light source with a wavelength of 465 nm and a power density of 34 mW/cm^2^ for 15 min, observed a dose-dependent induction of cytotoxicity, cell cycle arrest in the G0/G1 phase, apoptosis induction, and autophagy induction.

In the study by Zhang et al. [126], curcumin was tested on male nude (nu/nu) mice deficient in T cells with xenotransplanted A549 lung carcinoma tumors. Curcumin was administered as nanoparticles at an equivalent 15 mg/kg dose. Photodynamic therapy was performed by irradiating the mice with an LED light source. The observed effects included significant regression of the tumor, with a reduction in tumor volume of ~74% in the group treated with co-doped nanoparticles and irradiated compared to the control groups.

Prathyusha et al. [127] conducted in vitro studies on human mammary cell lines MCF-7 and HEK-293. The curcumin concentrations used in the study were 1, 2, 4, 8, 16, and 32 μg/mL irradiated with blue light (460 nm) for 15 min. Liposomes loaded with the molecule were synthesized to improve curcumin delivery. The study observed that liposomes showed a higher generation of ROS than free curcumin in HEK-293 and MCF-7 cells. The increase in ROS levels in cells induced macro-molecular and organelle damage, triggering apoptotic cell death. It was demonstrated that ROS generation is concentration-dependent, with increased ROS generation and increased curcumin concentration in liposomes.

An in vitro experiment was conducted by Shao et al. [128] on human lung cancer cell lines A549 and SPCA1. The study treated cells with a concentration of 20 μM curcumin for 12 h. This concentration was chosen as the maximum non-lethal dose for the cells. The irradiation parameters used were a wavelength of 425 nm and an intensity of 40 mW/cm^2^ for 60 s, corresponding to a total light dose of 2.4 J/cm^2^. The study’s observed effects of PDT with curcumin included the inhibition of epithelial–mesenchymal transition in lung cancer cells, the induction of autophagy, and a reduction in the migration and invasion of tumor cells.

Bechnak et al. [129], studying human lung adenocarcinoma (A549) and human malignant melanoma (A375) cells, highlighted that a minimum dose of curcumin of 1.8 ± 0.1 μM for A549 cells and 3.9 ± 0.1 μM for A375 cells in the form of nanocapsules and irradiation with blue light for a total of 30 min after a 6 h drug incubation period resulted in a significant increase in single- and double-strand DNA breaks in cells exposed to light.

On melanotic melanoma (A375) and amelanotic melanoma (C32) cell lines, human keratinocytes (HaCaT), and human fibroblasts (HGF), Szlasa et al. [130] found that concentrations of curcumin ranging from 5 μM to 50 μM and a wavelength of 410 nm induced increased cell death, increased expression of caspase-3, and DNA cleavage. Additionally, reduced cell proliferation was observed due to the rearrangement of the actin cytoskeleton. The minimum effective dose of curcumin in lowering melanoma cell viability was 5 μM.

Wozniak et al., [131] in an experiment conducted on three different cell lines, MUG-Mel2 (melanoma), SCC-25 (squamous cell carcinoma), and HaCaT (normal keratinocytes), with curcumin and irradiation parameters of a constant radiation power of 20 mW/cm^2^ for 2 min (2.5 J/cm^2^) with blue light (380–500 nm), were able to induce increased apoptosis in SCC-25 and MUG-Mel2 cells 24 h after the proposed therapy. SCC-25 cells showed increased apoptosis as the leading cause of cell death, while MUG-Mel2 cells showed both types of cell death as possible mechanisms.

Kazantzis et al. [132] conducted an experiment using the LNCaP prostate cancer cell line. Cells were treated with 3 μM curcumin for 1 h, followed by blue light at 360 mJ/cm^2^, inducing death in 40–50% of cells 24 h after irradiation.

He et al. [133], in a study on the cervical cancer cell line Me180 and in vivo through xenotransplanted mice, showed that a minimum dose of curcumin of 5 μmol/L and blue light irradiated at a dose of 100 J/cm^2^ for an irradiation time of 180 s induced apoptosis of Me180 cervical cancer cells and the3 suppression of tumor growth in vivo. In particular, treatment on xenotransplanted mice resulted in a significant reduction in Notch1 gene expression. Furthermore, histopathological analysis of xenotransplanted tissues revealed a reduction in tumor volume, a decrease in the nucleoplasmic ratio, the presence of pyknotic nuclei and perinuclear halos, as well as a decrease in mitotic figures compared to the control group. Notably, significant necrosis was observed in treated tumor tissues.

HeLa (human cervical cancer) and HT-29 (human colon adenocarcinoma) cell lines were exposed to a concentration of free or micelle-bound curcumin of 10 μg/mL and irradiated with a tungsten lamp at a power of 0.04 W/cm^2^ for various periods and showed effective inhibition of cell growth, with increased cell mortality observed with the use of micelles [134]. Jamali et al. [135], on the DKMG/EGFRvIII cell line derived from glioblastoma multiforme, were able to assess the intrinsic cytotoxic effect of blue light, curcumin, and poly(lactic-co-glycolic acid) nanoparticles containing curcumin. Finally, photodynamic therapy with curcumin is effective in oral squamous cell carcinoma cells, inhibiting cell proliferation and apoptosis induction. Beyer and colleagues [136] discovered that treating human head and neck squamous cell carcinoma (HNSCC) lines with curcumin concentrations between 0.027 μM and 2.71 μM for 1 h, followed by 5 min of exposure to either 1 J/cm^2^ UVA or a Philips visible light bulb (spectrum: 380–780 nm), resulted in decreased cell proliferation, increased ROS production, and enhanced DNA fragmentation. In a separate study, Dujic et al. [137] demonstrated that pre-treating cells with curcumin concentrations ranging from 0.677 μM to 5.42 μM for 1 h, followed by 5 min of exposure to either UVA (1 J/cm^2^) or a Philips visible light bulb (5500 lx, spectrum: 400–500 nm), yielded significant results. Specifically, combining 2.71 μM curcumin with visible light reduced cell proliferation to 17.3%, whereas the combination with UVA reduced it to 31.1%. Additionally, a decrease in metabolic activity was noted in cells treated with both curcumin and light, but not in those without light exposure. Notably, the combination with visible light led to a greater reduction in cell viability compared to UVA, resulting in 50% and 35% reductions, respectively [136].

## 4. Improving Curcumin’s Therapeutic Effectiveness: Applications of Nanotechnology for Anticancer Drug Delivery

**Figure 5:**
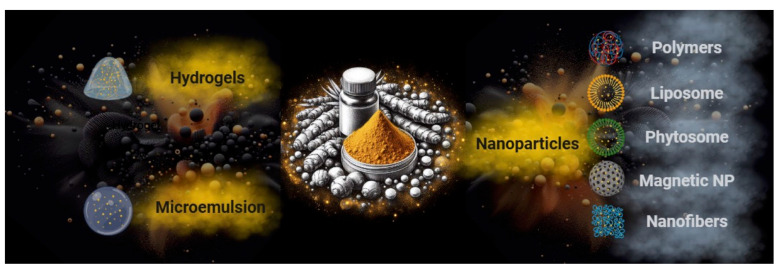
Improving curcumin’s therapeutic effectiveness: applications of nanotechnology for anticancer drug delivery. This illustration highlights various drug delivery systems developed to enhance curcumin’s bioavailability and therapeutic efficacy. These systems include hydrogels, microemulsions, nanoparticles (phytosomes, polymeric nanoparticles, liposomes, and magnetic nanoparticles), and implantable nanofibers. Each system offers unique advantages in terms of stability, targeted delivery, controlled release, and increased bioavailability, addressing the challenges posed by curcumin’s poor water solubility and rapid metabolism.

As described in the preceding paragraphs, while numerous studies highlight the therapeutic properties of curcumin and curcuminoids, bioavailability issues restrict their consistent clinical application. The challenge, therefore, lies in overcoming problems associated with its poor water solubility, rapid metabolism, oxidation, and hydrolysis in the gastrointestinal environment [138]. An approach to this challenge has involved exploring innovative solutions, such as drug delivery systems based on carrier composites [139]. Through this approach, it is theoretically possible to enhance the dissolution rate of poorly soluble molecules, improve adhesion to the intestinal mucosal membrane for prolonged residence time, facilitate greater drug permeation, and offer the advantage of bypassing first-pass metabolism by facilitating transport through M cells [140]. Carrier- and nanocarrier-based drug delivery systems developed for curcumin are represented by a broad family of formulations that can be mainly divided into hydrogels, microemulsions, and nanoparticles (Figure 5); however, the formulations may frequently be hybrid and not so categorizable. Implantable nanofibers, liposomes, phytosomes, and polymeric, magnetic, and solid nanoparticles have seen functional applicability for curcumin.

### 4.1. Nanoparticles

Nanoparticles, a key player in drug delivery, offer specific and targeted delivery of drugs. They shield drugs from degradation, provide structural stability, and enhance the bioavailability and specificity of administration. The types of nanoparticles vary based on their constituent components, offering a diverse range of options for drug delivery [140]. 

#### 4.1.1. Phytosomes

Phytosomes, a unique and advanced technology, enhance the delivery and efficacy of plant compounds. They do so by leveraging phospholipids’ biocompatibility to optimize active ingredient absorption [141]. The literature demonstrates that phytosomes can improve drug selectivity, allowing for a higher concentration of curcumin in cancer cells than in healthy cells. Phytosome carriers derived from soy lecithin/microcrystalline cellulose increased bioavailability 9-fold in patients given a dose of 367 mg curcumin [142]. A similar nanocarrier-based formulation further improved bioavailability by 12.7-fold [13]. In a mouse model, soy lecithin nanocarriers increased curcumin’s bioavailability at a dose of 250 mg/kg by 5.6-fold [143]. Applied to clinical anticancer research, the curcumin lecithin delivery system (Meriva^®^) alleviated the adverse effects of chemotherapy and radiotherapy in 160 patients. Phytosomal curcumin administration (500 mg/day) influenced the upregulation of antioxidative responses and reduced inflammatory pathways [144]. Meriva phytosomal curcumin, administered in two 1000 mg daily doses, improved all International Prostate Symptom Score items, except urinary incontinence, in both groups, with a trend toward greater efficacy of phytosomal curcumin and no adverse reactions [145]. Curcuminoids contained in Meriva^®^ administered as three 300 mg capsules (one capsule) per day induced the reduction in Tumor Necrosis Factor-alpha, Transforming Growth Factor-beta, interleukin-6, Substance P, high-sensitivity C-reactive Protein, Calcitonin Gene-Related Peptide, and Monocyte Chemoattractant Protein-1 in patients with a solid tumor [146]. Pure or phytosomal curcumin (Meriva^®^) was administered orally to mice with oral tumor lung metastases. Phytosomal curcumin caused a significant increase in the expression of Matrix Metalloproteinase-9 and inhibited lung metastasis [147].

#### 4.1.2. Polymers

Polymeric micelles are a promising drug delivery system designed to achieve different sizes and shapes. The system can protect the encapsulated drug from the harsh conditions in the gastrointestinal tract by facilitating controlled release at specific target sites. The mucoadhesive properties of the micelles increase dwell time and inhibit efflux pumps, leading to improved drug accumulation and efficacy [148]. The γ-cyclodextrin nanocarriers loaded with 207 mg curcumin increased the bioavailability of curcuminoids by 29.8-fold in patients [149]. A polymeric solid dispersion formulation loaded with 367 mg curcuminoids resulted in a 45.9-fold increase in human bioavailability [13]. Particles of polylactic-co-glycolic acid-polyethylene glycol with 50 mg/kg curcumin improved bioavailability by 55.9-fold in rats [150]. Also, in the mouse model, the increase was 9.3-fold when curcumin was administered in D-α-Tocopheryl polyethene glycol succinate (D-α-Tocopheryl polyethene glycol succinate) nanoparticles at a dosage of 75 mg/kg [151]. Chitosan or eudragit-coated chitosan particles used to administer 10 mg/kg of curcuminoids to rats resulted in a 2.1-fold and 3.6-fold higher bioavailability of curcumin [152]. Xie et al. [153], via polymeric nanoparticles, increased curcumin bioavailability in rats by 5.6-fold. Human mammary carcinoma tumor cells (MCF7) and normal mouse lung fibroblast cells (L929) were tested with nanoparticles composed of chitosan modified with folic acid and loaded with 0.5 mg curcumin. The results showed that curcumin-loaded nanoparticles significantly affected human mammary carcinoma tumor cells. However, they had the opposite effect on normal mouse lung fibroblast cells, increasing cell growth and proliferation [154]. Micelles consisting of a methoxy-polyethylene glycol-poly,l-lactide copolymer (mPEG-PLA) loaded with curcumin at a concentration of 11.06 ± 0.8% (*w*/*w*) were tested for their potential anticancer activity on B16F10 murine melanoma cells and MDA-MB-231 human breast adenocarcinoma cells. Doses of 50 and 100 μg/mL of free curcumin or curcumin–mPEG-PLA formulations were administered for 1 h and 4 h, respectively. Increased cellular uptake was observed. This resulted in increased cytotoxicity compared to free curcumin [11]. Micelles consisting of an amphiphilic polymer derivative of poly(beta-aminoester) and containing D-alpha-tocopheryl succinate/phosphatidylethanolamine as the main components were loaded with a drug load of 98.3 ± 1.92% and an encapsulation efficiency of 14.8 ± 0.16%. Their efficiency was tested in mice with Lewis lung carcinoma (3LL) and mammary carcinoma. A significantly higher ability to reverse multiple drug resistance was observed. The tumor size was also described [155]. Abruzzo et al. [156] prepared chitosan-based nanoparticles that could encapsulate lipophilic molecules and be loaded with curcumin. Curcumin could be delivered to colon cancer cells in vitro. Nanoparticles with or without curcumin were prepared with chitosan, hyaluronic acid, and sulfobutyl-ether-β-cyclodextrin. The optimized formulations led to a reduction in tumor cell proliferation. This was due to the increased expression of genes involved in apoptosis. A self-assembling structure of curcumin–cyclodextrin using inclusion complexes was developed by Yallapu et al. [157] in DU145 prostate cancer cell lines, and the optimized inclusion complex was evaluated for increased intracellular uptake and anticancer activity. Compared to free curcumin, an increased cellular uptake of curcumin was reported. The micelles were shown to have potent anticancer effects through the induction of reactive oxygen species production, preferential uptake into tumor cells, and improved magnetic resonance imaging properties for better tumor targeting. Shahriari et al. [158] highlighted the antitumor effects of curcumin-loaded polymeric nanoparticles in a literature review; curcumin-loaded nanoparticles showed significant cytotoxicity in the SCC25, MDA-MB-231, and A549 cell lines, with a decrease in tumor cell proliferation, an increase in ROS, and an increase in apoptosis.

#### 4.1.3. Liposomes

Liposomes are small, spherical, synthetic vesicles that can be made from cholesterol and natural phospholipids. They are non-toxic and immunogenic. They are flexible, biocompatible, and biodegradable. Through the incorporation of drugs, they provide good thermal stability and solubility. They are both hydrophobic and hydrophilic. These unique properties of liposomes make them suitable as drug delivery systems [159]. Liposomes of curcumin coated with thiol-derived chitosan achieve a drug encapsulation efficiency of 94% and exhibit more excellent stability at room temperature and pH than uncoated liposomes, demonstrating the feasibility of targeted curcumin delivery to the cancer cell line [160]. Similarly, Cuomo et al. [161] developed chitosan-coated liposomes loaded with curcumin. These liposomes can increase the bioavailability of curcumin compared to free curcumin. When liposomes loaded with curcumin were administered to Sprague-Dawley rats at a dose of 100 mg/kg, the bioavailability of the molecule was increased by a factor of 5 [162]. Using uncoated liposomes and Trimethylchitosan-coated liposomes to deliver 40 mg/kg of curcumin to Wistar rats resulted in a 2-fold increase in bioavailability, as reported by Chen et al. [163]. Curcumin-loaded salbutamol liposomes were tested on the human neuroblastoma BCI-NS1.1 cell line. The anti-inflammatory properties of the liposomes were evaluated. The liposomes reduced the levels of several proinflammatory markers, including interleukin-8, -6, and -1β, and Tumor Necrosis Factor-alpha [164]. In lung cancer, curcumin liposomes’ effect on the Lewis LL/2 lung carcinoma cell line in mice showed the ability to block LL/2 cells in the G2/M phase. This indicates a potential anticancer effect [165]. Furthermore, improved cell delivery and superior anticancer efficacy were demonstrated using curcumin–polyethyleneglycol–polyethyleneimine liposomes (200 μg/mL of curcumin) on lung carcinoma A549 cells [166]. Curcumin liposomes with β-cyclodextrin showed better inhibition of A549 cells, higher cytotoxicity, and fewer side effects [167]. In cervical cancer, curcumin-loaded cationic liposomes on cervical cancer Hela and SiHa cells increased cell apoptosis and enhanced cytotoxicity [168]. In addition, curcumin-loaded β-cyclodextrin liposomes on Hela cells showed additional stability and cellular delivery. They protected against leakage and provided a longer retention time with higher cytotoxicity [169]. In prostate cancer, curcumin liposomes on PC-3 human cancer cells promoted the drug’s uptake, with a superior concentration- and time-dependent inhibitory effect and targeting activity [170]. In addition, nanoliposomes of curcumin on human prostate cancer LNCaP and C4-2B cells improved the bioavailability and the anticancer effect [171]. In breast cancer, curcumin nanoliposomes on MCF-7 cells showed dose-dependent inhibition of cell cycle arrest and induction of apoptosis. Its bioavailability was also improved [172]. Liposomes of curcumin–γ-cyclodextrins on MCF-7 cells have been shown to have superior antitumor activity with fewer side effects [173].

#### 4.1.4. Magnetic Nanoparticles

Magnetic nanoparticles offer several key advantages in biomedical applications, making them extremely attractive for various purposes [174]. External magnetic fields can remotely control magnetic nanoparticles, enabling targeted drug delivery, magnetic separation, and manipulation within the body. In addition, magnetic nanoparticles exhibit negligible background signals in biofluidic samples and biological tissues. This reduces interference with magnetic signals in biosensing and imaging applications [174,175]. Magnetic nanoparticles can be used in hyperthermic magnetic therapy. Their dynamic magnetization under alternating magnetic fields makes them effective tools in cancer treatment. Magnetic nanoparticles exhibit greater reactivity and versatility than bulk materials due to their high surface-to-volume ratio. In addition, magnetic nanoparticles can be designed with surface modifications to improve their biocompatibility [176]. This makes them suitable for various biomedical applications [174]. Iron oxide (Fe_3_O_4_), magnetite, or other magnetic materials can form these nanoparticles. Curcumin-loaded Fe_3_O_4_ nanoparticles coated with L-tyrosine may provide a versatile transport system for potentially delivering curcumin [177]. Magnetic nanoparticles of c-Fe_2_O_3_ (iron oxide) functionalized with curcumin via the biodegradable polymer chitosan enhanced controlled and pH-sensitive curcumin release [178]. Magnetic nanoparticles based on poly(lactate-co-glycolate) loaded with paclitaxel and curcumin enabled curcumin to cross the blood–brain barrier for targeted drug delivery to brain tumors [179]. In a recent review, Rezae et al. [176] highlighted how combining magnetic nanoparticles and curcumin offers new opportunities for improved biocompatibility, precise targeting, controlled drug delivery, and innovative therapeutic applications such as hyperthermic magnetic therapy in treating cancer.

Iron oxide nanoparticles decorated with folic acid and loaded with curcumin were tested on cervical cancer, specifically on HeLa cells and L929 fibroblasts. The nanoparticles were shown to be tumor-targeting with increased selectivity, allowing for targeted uptake of curcumin into the tumor. The viability of HeLa tumor cells was reduced over time due to the high cytotoxicity. The nanoparticles increased T2 signal intensity on MRI. This facilitated the diagnosis of cervical cancer cells. A low level of toxicity and transient effects were observed in fibroblasts [180]. Fe_3_O_4_ nanoparticles coated with carboxymethyl chitosan containing curcumin in combination with hyperthermia significantly reduced the metabolic activity of breast cancer cells and induced cell death [181]. In addition, the encapsulation of curcumin in Polylactic acid–hyaluronic acid/Fe_3_O_4_ nanoparticles significantly increased toxicity toward colon cancer cells (HCT116) [182].

#### 4.1.5. Implantable Nanofibers

Nanofiber systems are promising for effectively delivering curcumin. Curcumin-loaded polylactic acid nanofibers are prominent among the various nanofiber systems used. Studies using water uptake, percentage porosity, morphology, cytotoxicity, and in vitro drug release have demonstrated controlled drug release from these nanofibers [183]. Furthermore, polylactic-co-glycolic acid nanofibers loaded with curcumin have shown efficacy in treating tumors, with a high drug encapsulation efficiency and sustained release without initial bursting [184]. Another interesting option is using mesoporous silica nanoparticles embedded in polylactic-co-glycolic acid to sustain curcumin’s release [185]. These nanoparticles can improve the drug’s bioavailability. Due to their small size, they can accumulate at the tumor site due to enhanced permeation and retention effects. Thangaraju et al. [184] worked on fabricating a Poly-L-Lactic acid scaffold loaded with curcumin and reported controlled drug release by evaluating several parameters such as water absorption, percentage porosity, morphology, cytotoxicity, and in vitro drug release. Thuy et al. [186] conducted an in vivo experiment on nanofibers and developed a nanofiber patch for wound healing. The curcumin-loaded Poly-L-Lactic acid nanofibers had an estimated mean diameter of 562 nm with a range of 300–1200 nm and pores on the surface of the nanofibers, probably due to the use of a mixture of the volatile solvent dichloromethane and the non-volatile solvent N,N-dimethylacetamide. These pores on the nanofibers’ surface can increase the surface area. This could promote cell adhesion, enhance controlled drug release, and promote wound healing.

In addition, curcumin-loaded xanthan–chitosan nanofibers and curcumin-loaded zein–silk–chitosan nanofibers have been proposed as active wound dressings. They have significant potential in promoting skin wound healing [187]. Other approaches include curcumin-loaded Bombyx mori silk nanofibers, which have a high porosity and water absorption capacity, making them suitable for drug delivery [188]. Furthermore, under both saliva-stimulated and gastrointestinal-stimulated conditions, almond gum/polyvinylpyrrolidone nanofibers loaded with a curcumin–b-cyclodextrin complex demonstrated good drug loading performance and a significant release profile [189]. Overall, these innovative delivery systems demonstrate significant advantages over conventional drug delivery. They improved curcumin’s therapeutic efficacy, stability, and bioavailability for various medical applications.

In the study by Xie et al. [153], a curcumin–silk fibroin nanofibrous matrix was used to study solid tumors formed by HCT-116 cells in vivo in BALB/c nude mice. Curcumin silk fibroin nanofibrous matrices showed improved intracellular uptake, enhancing anticancer activity.

Cheng et al. [190] implanted a curcumin/gelatin nanofibrous matrix on pancreatic adenocarcinoma in wild-type mice. They showed that topical application suppresses the growth of the xenografted tumor. A breast cancer cell line (MCF-7 cells) treated with a curcumin–polycaprolactone/Poly(amidoamine) nanofibrous scaffold showed high cell death rates [191].

Sedghi et al. [192] used curcumin-loaded silk fibroin (S4)/Polyvinyl alcohol nanofibers in breast cancer models (normal fibroblastic cells and 4T1 tumor cells) that inhibited tumors without showing cytotoxicity to normal cells. They also induced increased proliferation of healthy cells. Curcumin-loaded poly-L-lactide nanofibers showed a 60–80% inhibitory effect on C6 glioma cells, compared to 15–25% in the normal cell line (NIH 3T3 fibroblasts). Curcumin stimulated the growth of fibroblasts at low doses, while it inhibited their growth at high doses [184].

Guo et al. [193] observed in a study on glioma 9L that poly(ε-caprolactone)-poly(ethylene glycol)-poly(ε-caprolactone) (PCEC) fibers affected the tumor without cytotoxicity to healthy cells.

### 4.2. Microemulsions

Microemulsions are isotropic and thermodynamically stable systems composed of oil, water, surfactants, and often co-surfactants. Particle sizes range from 20 to 200 nm [194]. A microemulsion denotes a classification based on thermodynamic stability and composition rather than size, although droplet sizes can be nanometric. These microemulsions can encapsulate hydrophilic and hydrophobic drugs, protecting them against enzymatic hydrolysis and oxidation. They can also enhance the solubility of lipophilic drugs, thereby improving their bioavailability [194,195]. Oil-in-water emulsions using sodium caseinate as an emulsifier have been shown to improve the solubility of curcumin. This improves its oral delivery as a drug [196]. Similarly, an emulsion containing casein and soy polysaccharides improved curcumin’s solubility, stability, and bioavailability [197]. Sophorolipid-coated nanoparticle microemulsions increased the bioavailability of curcumin (dose 100 mg/kg) by 3.6-fold in Sprague-Dawley rats [198]. In the same model, the bioavailability of 200 mg/kg of curcumin was increased by 22.6-fold in a microemulsion containing Capryol 90 (oil), Cremophor RH40 (surfactant), and Transcutol P aqueous solution as a co-surfactant [199]. Ochoa-Flores et al. [200] showed that the bioavailability of curcumin (dose 50 mg/kg) in BALB/c mice was improved by 188-fold by using a nanoemulsion containing phosphatidylcholine and phosphatidylcholine enriched with medium-chain fatty acids (42.5 mol%) combined with glycerol as a co-surfactant. In Sprague-Dawley rats, the bioavailability of curcumin (20 mg/kg) was increased by 7.9-fold using Conventional Self-Double and Amorphous Solid Dispersion curcumin nanoemulsions [201].

Recent advances in encapsulating curcumin in nanoemulsions include techniques such as phase inversion temperature, phase inversion composition, ultrasonication, high-pressure homogenization, and microfluidics [202]. These methods control the droplet size after incorporation and play a crucial role in improving curcumin’s loading efficiency and encapsulation. Furthermore, in designing effective nanoemulsion systems with enhanced stability and bioavailability, factors such as emulsifier type and concentration, oil type and volumetric fraction, and emulsifier–curcumin interactions are fundamental [203]. Chen et al. [204] demonstrated significant effects on the cell cycle, apoptosis, and expression of key proteins in HT-29 colon cancer cells using poly(lactate-co-glycolate) microemulsions and liposomal formulations containing 5 to 40 µM of curcumin. The treatments induced cell cycle arrest in the S phase. The microemulsion showed a higher proportion of apoptotic cells compared to the free curcumin extract. Both treatments led to a dose-dependent decrease in viable and necrotic cells. There was a dose-dependent increase in early and late apoptotic cells. Both treatments resulted in a dose-dependent increase in the expression of key proteins such as cytochrome C, providing strong evidence of the efficacy of microemulsions in drug delivery. Peng et al. [205] observed significant inhibition of tumor growth, necrosis, apoptosis, and the suppression of proliferation compared to free curcumin in BALB/c nude mice bearing Hep G2 hepatocarcinoma tumors using nanogels loaded with curcumin at a concentration of 20 mg per kg body weight. Guerrero et al. [206] used a curcumin nanoemulsion system consisting of curcumin, Miglyol 812, Epikuron 145 V, acetone, ethanol, and Milli-Q water. The curcumin dose was 1500 μM. The study was carried out in C57BL/6 mice using B16F10 melanoma cells. It demonstrated the efficacy of the nanoemulsion in preventing tumor recurrence and metastasis. Using MCF7 (human breast cancer) or HepG2 (human liver cancer) cancer cells and Human Umbilical Vein Endothelial or Human Embryonic Kidney 293 cells, Notarbartolo et al. [207] demonstrated that curcumin microemulsions exhibit high antitumor activity. In particular, compared to free curcumin, curcumin nanoemulsions induced higher cytotoxicity in MCF7 cells. A synergistic effect was observed in tumor cells when curcumin was combined with cisplatin. In addition, Ombredane et al. [208] reviewed that curcumin nanoemulsions exhibited high antitumor activity in liver and breast cancer. Malignant tumor cells showed greater sensitivity compared to normal cells.

### 4.3. Hydrogels

Hydrogels are three-dimensional networks of hydrophilic polymers with a high capacity for water retention. They are used for drug delivery. They can release active ingredients in a controlled manner. Their porosity, as well as their response to pH and temperature, can be adjusted. Hydrogels can be designed to be biocompatible and biodegradable [209]. In terms of size, hydrogels are not typically classified as nanocarriers. Their structures are often much more significant (typically microscopic rather than nanoscopic). However, there are “nanogels”. These are a subclass of hydrogels designed with nanoscale dimensions and can be considered nanocarriers.

Hydrogels have improved the delivery and biostability of curcumin molecules. Efficacy against inflammatory processes and bacterial infections has been demonstrated with curcumin hydrogels based on polyvinylpyrrolidone and poloxamers [210]. Polyvinyl alcohol and borax (sodium borate) hydrogels have been shown to have potential in wound healing and tissue regeneration [211].

The use of hydrogels for curcumin delivery was evaluated against emulsions and aqueous solutions in a review by Zheng et al. [212]. They identified several advantages: Hydrogels can provide more excellent curcumin protection than aqueous solutions, as curcumin is entrapped within the microgel structures. Hydrogels can provide more excellent chemical stability for curcumin compared to emulsions and aqueous solutions, particularly at neutral pH. Hydrogels can be used in highly viscous and opaque products. This offers flexibility in incorporating curcumin into different types of products. However, some drawbacks have been noted: Hydrogels can promote curcumin degradation at both acidic and neutral pH, particularly when curcumin is encapsulated in alginate beads. Hydrogels can quickly sediment in low-viscosity and transparent products, limiting their applicability in certain products.

As highlighted in a recent review by Stachowiak et al. [213], hydrogels can be designed to release curcumin in a controlled manner, allowing for prolonged and targeted therapeutic action. However, their true potential lies in incorporating multiple therapeutic agents alongside curcumin. This opens up possibilities for synergistic effects, potentially revolutionizing treatment efficacy.

As demonstrated by Hussein et al. [214], the success of curcumin-loaded Polyvinyl alcohol/cellulose nanocrystal hydrogel membranes in cancer treatment is a beacon of hope in the field. These membranes, with their prolonged release profile of curcumin, offer better bioavailability and prevent rapid metabolism and elimination from the bloodstream. The curcumin in the membranes showed cytotoxic effects on breast MCF-7 and liver Huh-7 cancer cells while not significantly affecting the viability of normal cells. The membranes effectively inhibited cancer cell proliferation through inducing apoptosis and influencing cell cycle progression, marking a significant step forward in cancer treatment.

## 5. The Curcumin–Drug Interaction: A Mixed Blessing

**Figure 6:**
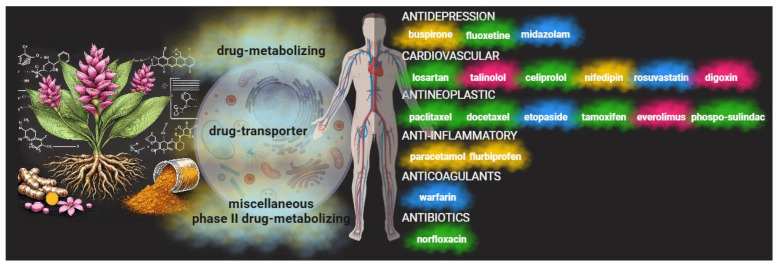
Curcumin and drug interactions. Curcumin has demonstrated an ability to interact with cellular pathways such as cytochrome P450 and other drug transporters. These play a key role in modulating the pharmacokinetics of conventional drugs. Consequently, through synergistic or antagonistic behavior, curcumin can influence a drug’s efficacy. In some cases, there is no discernible effect (yellow clouds); in others, the effects are mixed and concentration-dependent (blue clouds). Lastly, curcumin can support the drug effects (green clouds) or be potentially dangerous (red clouds). Thus, the therapeutic activities of various categories of drugs used in antitumor therapy, in managing tumor-related side effects, or in specific therapies can be altered.

Conventional medicine derives most of its drugs from natural compounds, which undergo chemical modifications to enhance their safety and efficacy. On the other hand, phytopharmaceuticals are botanical formulations containing purified plant materials or extracts commonly used in their natural state and readily available as dietary supplements [215]. However, it is crucial to consider that phytopharmaceuticals can interact with conventional drugs, much like traditional drugs [216]. Recent research by Choi et al. [217] has highlighted potential interaction effects of phytopharmaceuticals on the metabolism of drugs such as warfarin, cyclosporine, antihypertensive agents, oral contraceptives, and neurological drugs. For instance, pathways like cytochrome (CY) P450 and other drug transporters play a crucial role in altering the pharmacokinetics of conventional medications when combined with herbal supplements, as demonstrated in a study by Wang on patients with cardiovascular issues [218]. Garlic, which possesses anticoagulant properties, may induce bleeding when combined with warfarin [219]. The prescription of St. John’s wort used as an antidepressant may alter oral contraceptives, immunosuppressants, lipid-lowering agents, other antihypertensive drugs, and even chemotherapeutic agents [220]. As described in the preceding paragraphs, the literature data on curcumin arouses interest in its potential therapeutic properties. Therefore, it is crucial to consider the interactions that curcumin might have with other drugs, as these interactions can affect the efficacy and safety of pharmacological treatment (Figure 6).

Curcumin’s interactions with other medications can be complex and depend on various factors, including dosage, treatment duration, and individual patient characteristics. Its anti-inflammatory and antioxidant properties may positively and synergistically interact with some cardiovascular, antidepressant, antimicrobial, and chemotherapeutic drugs, enhancing their therapeutic effects. On the other hand, curcumin’s interactions with drugs may lead to increased side effects and decreased pharmacological efficacy [215,216,219].

As highlighted in a review by Bahramsoltan et al. [216], curcumin may influence drug metabolism and the activity of drug-metabolizing enzymes and transporters such as CYP450 and drug transporter P-glycoprotein (P-gp), as well as various phase II drug metabolizers. In particular, curcumin may inhibit several isoforms of CYP enzymes, which play a crucial role in the metabolism of many drugs. Curcumin’s inhibition of CYP enzymes may lead to altered metabolism and clearance of co-administered drugs, potentially affecting their efficacy and safety. For example, in a comprehensive study by Appiah-Opong et al. [221], curcumin is described as a competitive inhibitor of CYP1A2, with an IC50 value of 40.0 μM. However, Bahramsoltan et al. [216] suggest conflicting reports on the exact IC50 value of curcumin for CYP1A2, with some studies showing a higher value. Demethoxycurcumin, another compound present in turmeric, also inhibits CYP1A2 [216]. Furthermore, curcumin and curcuminoid extract are competitive inhibitors of the CYP2B6 enzyme, with an IC50 of 24.5 μM [221]. Curcumin affects CYP2C9 as a non-competitive inhibitor, with an IC50 of 4.3 μM [221]. Additionally, it inhibits CYP2C19, responsible for metabolizing drugs like clopidogrel. Curcumin acts as a non-competitive inhibitor of CYP2D6, with variable IC50 values reported in different studies [216,221]. It is important to note that curcumin’s effect on CYP activity, such as that of CYP2D6, may vary depending on the experimental model used.

The interaction of curcumin with P-gp and other drug-metabolizing enzymes is crucial for understanding its potential effects on drug metabolism and bioavailability. Curcumin can inhibit P-gp activity at various concentrations, influencing expression and reducing the activity of multidrug resistance protein 1 (MDR1) levels [222,223]. Additionally, curcumin affects uridine diphosphate glucuronosyltransferase, essential for glucuronidation reactions in drug metabolism [224]. Curcumin inhibits sulfotransferase activity, which transfers a “sulfur” moiety to xenobiotics, as well as glutathione-S-transferase enzymes, which play a role in detoxifying electrophilic substrates [225]. These interactions could result in altered pharmacokinetics that may lead to variations in the effects of antineoplastic drugs and drugs that can be used in managing the side effects of antitumor therapy and the disease itself.

Curcumin has shown a positive impact by enhancing the effects of various antineoplastic agents. The co-administration of curcumin with paclitaxel led to a significant increase in the Area Under the Concentration–Time Curve (AUC) and the bioavailability of paclitaxel, along with the increased accumulation of paclitaxel in tumor tissue [226]. Curcumin increased the AUC and reduced the clearance of docetaxel significantly, suggesting a potential enhancement of this drug’s efficacy [227]. It also significantly increased the AUC, Cmax, and bioavailability of etoposide due to decreased intestinal P-gp and CYP3A4 activity [228]. However, the effect of curcumin on intravenously administered etoposide was insignificant. Curcumin altered the pharmacokinetics of tamoxifen by reducing CYP3A4 and P-gp activity, resulting in increased Cmax and AUC [229]. Curcumin influenced the pharmacokinetics of phospho-sulindac by increasing the Cmax and AUC, both in solubilized and nanoparticulate forms. This interaction could positively impact the anticancer activity of phospho-sulindac. However, it is crucial to consider that P-gp and CYP inhibition could also lead to an increased risk of toxicity or adverse effects [230]. Finally, the co-administration of curcumin with everolimus resulted in a significant decrease in the AUC and Cmax of everolimus, which could negatively influence its therapeutic efficacy and increase the risk of side effects [231].

Interactions with antidepressant drugs have also been described with midazolam. In the case of rats, oral administration of curcumin increased the AUC of midazolam and reduced its clearance, primarily through the downregulation of the intestinal CYP3A4 isoform. However, a randomized controlled study on healthy volunteers showed no significant changes in midazolam pharmacokinetics with short-term curcumin treatment [232]. Additionally, while curcumin enhanced the antidepressant effects of fluoxetine in mice, no changes were observed in serum and brain levels of fluoxetine when administered with curcumin, indicating the absence of pharmacokinetic interaction [233]. However, a study conducted on Sprague-Dawley rats showed that the co-administration of curcumin with buspirone did not significantly change buspirone pharmacokinetic parameters [234].

Curcumin showed a supportive effect during cardiovascular drug therapy. Oral administration of curcumin resulted in a 3.5-fold increase in losartan’s Cmax and a 1.7-fold increase in the AUC. Additionally, curcumin significantly altered the pharmacokinetics of EXP3174, the active metabolite of losartan, with 3.2-fold increases in the Cmax and 1.9-fold increases in the AUC [235]. The administration of curcumin for four days with celiprolol in rats caused a significant increase in celiprolol’s Cmax and AUC and a 22% reduction in celiprolol’s clearance [232]. The co-administration of curcumin with rosuvastatin increased rosuvastatin’s Cmax, AUC0-∞, and AUC0–24 in animal models. This interaction is mediated by the inhibition of OATP transporters by curcumin metabolites. However, since statin-associated myopathy is a critical dose-dependent side effect, increased serum levels of these drugs could lead to increased adverse effects [236]. Negative effects were described in a study on healthy volunteers, where curcumin administered at 300 mg per day with a single dose of talinolol significantly reduced talinolol’s Cmax and AUC and increased talinolol’s clearance. Excessive activation of multidrug resistance-associated protein 2 in response to curcumin, which inhibits P-gp, is hypothesized to be involved in this pharmacokinetic alteration [237]. Curcuminoids in turmeric can inhibit P-glycoprotein, a transporter involved in digoxin transport through the intestine. This could increase plasma digoxin concentrations and potentially increase the risk of digoxin toxicity [238]. Finally, a study on healthy volunteers showed that the combination of turmeric extract and nifedipine did not have significant interactions [239].

Regarding a possible effect of curcumin on anticoagulant and anti-inflammatory drugs, studies have shown that the co-administration of curcumin and warfarin for seven days resulted in a 1.5-fold increase in warfarin’s Cmax and a 1.6-fold increase in the total warfarin AUC, with a 57.14% reduction in warfarin’s clearance. Despite these pharmacokinetic changes, it has been reported that warfarin’s pharmacodynamic parameters, such as anticoagulant activity, were not altered during the experiment. However, since warfarin has a narrow therapeutic window and even small increases in its serum level can cause bleeding and subsequent complications, it is essential to carefully monitor the co-administration of curcumin and warfarin [240]. The co-administration of curcumin with paracetamol and flurbiprofen showed no significant changes in pharmacokinetics [241]. Regarding antibiotics, the co-administration of norfloxacin with curcumin increased the AUC and other pharmacokinetic parameters, with a reduction in the required norfloxacin dose [242]. Prolonged use of this combination may need to be evaluated, as it could increase the adverse effects of norfloxacin.

Finally, considering the critical role of curcumin in the prevention and treatment of diabetes and its associated disorders, it is essential to consider that curcumin could favorably affect most of the leading aspects of diabetes, including insulin resistance, hyperglycemia, hyperlipidemia, and islet apoptosis and necrosis. Therefore, it is essential to carefully monitor blood sugar levels or other blood parameters if taking diabetes medications [243].

## 6. Outlook for the Future: Curcumin Combination Chemotherapy

The unique properties of curcumin and its potential interactions with other drugs have led to the emergence of combination chemotherapy with curcumin. This innovative approach, explored across various cancer types, has shown significant promise. Notably, it has been found to boost therapeutic effectiveness while reducing adverse effects. For instance, it can counteract oxidative stress, a pivotal factor in the onset of several cancers. This stress is triggered by the excessive production of ROS, harmful by-products of metabolic processes, and immune responses. High ROS levels result in substantial cellular damage, including DNA mutations, lipid peroxidation, and protein oxidation, all of which contribute to cancer initiation and progression [244]. Lipid peroxides, hypochlorite, hydroxyl radicals, singlet oxygen, hydrogen peroxide, hypochlorous acid, and superoxide anions are among the primary forms of ROS involved in cancer. These reactive molecules interact with cellular components, disrupt cellular functions, and promote oncogenic transformations [245]. The chronic presence of ROS leads to the sustained activation of inflammatory pathways through the transcription factor NF-κB, which plays a central role in promoting inflammation and carcinogenesis [246]. Inflammation, a natural response to injury or infection, can become a significant risk factor for certain cancers when they become chronic. This is particularly true for colon and pancreatic cancer. Chronic inflammatory responses can ramp up cell proliferation, survival, and angiogenesis, while inhibiting apoptosis, all of which are key features of cancer. Transcription factors like NF-κB and STAT3, inflammatory enzymes such as COX-2 and MMP-9, and pro-inflammatory cytokines like IL-1, IL-6, IL-8, and TNF-α are crucial in inflammation-induced cancer. Understanding and targeting these mediators could offer new avenues for cancer treatment [246,247].

The IL-6/STAT3 signaling pathway is also essential in inflammation-associated cancers. These include liver and stomach cancer. IL-6 activates STAT3, which promotes the transcription of genes involved in cell survival and proliferation. Increased activity of IL-6 and STAT3 is associated with a poor prognosis in several types of cancer, including colorectal cancer [248]. Persistent activation of these pathways promotes tumorigenesis and contributes to chemo- and radioresistance, making cancer treatment difficult [249]. Curcumin may reverse cancer progression through the inhibition of IL-6R/STAT3 [250]. It also sensitizes cancer to antitumor and antimetastatic effects by suppressing the NF-kappa B cell signaling pathway [251].

Therefore, curcumin is an attractive candidate for combination chemotherapy due to its broad spectrum of biological activities. Its anti-inflammatory, antioxidant, and antitumor activities improve the efficacy of conventional chemotherapeutic drugs while reducing side effects [252].

In colorectal cancer, curcumin has been shown in vitro to sensitize cancer cells to 5-fluorouracil (5-FU) and oxaliplatin. Studies have shown that curcumin enhances the cytotoxic effects of these drugs by modulating several signaling pathways, including NF-κB and STAT3, leading to increased apoptosis and the inhibition of cell proliferation, migration, and invasion [253,254]. A synergistic effect in overcoming drug resistance was demonstrated by Howells et al. [255], who found that curcumin enhanced resistance to oxaliplatin chemo induction in both in vitro and in vivo colon cancer cells.

Tian et al. [256] showed that curcumin potentiates the antitumor effects of 5-FU in esophageal squamous cell carcinoma cells by downregulating NF-kappa B signaling, leading to increased apoptosis both in vitro and in vivo. Hartojo et al. [257] reported complementary mechanisms of apoptotic pathway activation, stating that the combination of curcumin and cisplatin had an additive effect in inducing apoptosis in esophageal adenocarcinoma cells.

Curcumin showed synergistic effects with paclitaxel and doxorubicin in breast cancer cell lines. Farghadani and Naidu [56], Mohammadian et al. [258], and Vinod et al. [259] found that the combination of curcumin and these chemotherapeutic agents downregulated HER2 and EGFR, which are often overexpressed in breast cancer, inhibited cell proliferation, and induced apoptosis. Curcumin sensitizes breast cancer cells to 5-FU by modulating signaling events involved in cell survival and apoptosis.

In clinical trials [260,261], patients with advanced breast cancer who received curcumin combined with docetaxel showed higher response rates compared to docetaxel alone. The combination was well tolerated and resulted in fewer side effects. This suggests the potential of curcumin to improve the efficacy of chemotherapy.

The combination of curcumin and gemcitabine significantly reduced tumor growth compared to either agent alone in a mouse model of pancreatic cancer. The combination therapy worked by inhibiting angiogenesis and inducing apoptosis by suppressing the NF-κB and COX-2 pathways [262]. Yoshida et al. [263] showed that curcumin can sensitize human pancreatic cancer cells to gemcitabine, potentially overcoming gemcitabine resistance.

Treatment with a combination of curcumin and cisplatin significantly reduced tumor size and weight in a mouse xenograft model of ovarian cancer [264]. The combination therapy was more effective than either agent alone. Also, it attenuated cisplatin-induced nephrotoxicity, highlighting curcumin’s protective role against chemotherapy’s side effects.

A phase II clinical trial in patients with metastatic colorectal cancer showed that adding curcumin to the standard FOLFOX regimen (5-FU, leucovorin, and oxaliplatin) improved overall survival and progression-free survival compared to the standard regimen alone [265].

Therefore, combining curcumin with chemotherapeutic agents in vitro and in vivo has shown a synergistic effect in cancer therapy.

As with the single molecule, using nanotechnology and drug delivery to combine curcumin with anticancer agents may offer significant advantages in cancer therapy, including enhanced synergistic effects, increased selectivity toward cancer cells, and reduced side effects. As expressed in a recent systematic review, in vitro studies have shown that combining curcumin with other anticancer agents in nanocarriers is feasible and can have a synergistic and even more targeted effect in destroying tumor cells [266].

## 7. Conclusions

In summary, curcumin demonstrates substantial therapeutic potential in cancer treatment [267] due to its multifaceted biological activities, including antioxidant, anti-inflammatory, and anticancer properties, exerting its potential therapeutic effects against various types of cancer [268,269,270]. For example, curcumin exhibits substantial anticancer properties particularly against gastrointestinal cancers, such as colorectal and pancreatic cancer, by inducing apoptosis, inhibiting cell proliferation, and modulating multiple cell signaling pathways [271,272]. Additionally, studies have shown promising results in the treatment of breast cancer, where curcumin interferes with cancer cell growth and metastasis [273]. The compound has also demonstrated efficacy in prostate cancer by targeting androgen receptor signaling [274] and in head and neck cancers through the inhibition of tumor growth and angiogenesis [275]. However, its clinical application is significantly hindered by its poor bioavailability, rapid metabolism, and low absorption [268]. Recent advancements in drug delivery systems, such as curcumin-loaded nanoparticles and hydrogels, have shown promise in overcoming these limitations, enhancing bioavailability, and ensuring sustained therapeutic efficacy [276]. Additionally, the integration of curcumin in photodynamic therapy has exhibited encouraging results, particularly in improving apoptosis and reducing tumor viability in vitro [277,278]. Despite these advances, further research is essential to optimize delivery mechanisms and fully understand curcumin’s pharmacokinetics and interactions with conventional drugs, especially given its potential to alter pharmacokinetic parameters and influence drug efficacy. Consequently, while curcumin holds great promise as an adjunct in cancer therapy, carefully considering its interactions and the continued development of innovative delivery systems is crucial for its successful clinical translation.

## References

[1] Sharifi-Rad J., Rayess Y.E., Rizk A.A., Sadaka C., Zgheib R., Zam W., Sestito S., Rapposelli S., Neffe-Skocińska K., Zielińska D. (2020). Turmeric and Its Major Compound Curcumin on Health: Bioactive Effects and Safety Profiles for Food, Pharmaceutical, Biotechnological and Medicinal Applications. Front. Pharmacol..

[2] Urošević M., Nikolić L., Gajić I., Nikolić V., Dinić A., Miljković V. (2022). Curcumin: Biological Activities and Modern Pharmaceutical Forms. Antibiotics.

[3] Rodrigues J.L., Prather K.L.J., Kluskens L.D., Rodrigues L.R. (2015). Heterologous Production of Curcuminoids. Microbiol. Mol. Biol. Rev..

[4] Ramirez-Ahumada M.d.C., Timmermann B.N., Gang D.R. (2006). Biosynthesis of Curcuminoids and Gingerols in Turmeric (*Curcuma longa*) and Ginger (*Zingiber officinale*): Identification of Curcuminoid Synthase and Hydroxycinnamoyl-CoA Thioesterases. Phytochemistry.

[5] Kriplani P., Guarve K. (2020). Physicochemical and Biological Aspects of Curcumin: A Review. Nat. Prod. J..

[6] Fuloria S., Mehta J., Chandel A., Sekar M., Rani N.N.I.M., Begum M.Y., Subramaniyan V., Chidambaram K., Thangavelu L., Nordin R. (2022). A Comprehensive Review on the Therapeutic Potential of *Curcuma longa* Linn. in Relation to Its Major Active Constituent Curcumin. Front. Pharmacol..

[7] Ayati Z., Ramezani M., Amiri M.S., Moghadam A.T., Rahimi H., Abdollahzade A., Sahebkar A., Emami S.A. (2019). Ethnobotany, Phytochemistry and Traditional Uses of *Curcuma* spp. and Pharmacological Profile of Two Important Species (*C. longa* and *C. zedoaria*): A Review. Curr. Pharm. Des..

[8] Rahmat E., Lee J., Kang Y. (2021). Javanese Turmeric (*Curcuma xanthorrhiza* Roxb.): Ethnobotany, Phytochemistry, Biotechnology, and Pharmacological Activities. Evid. Based Complement. Altern. Med..

[9] Priyadarsini K.I. (2014). The Chemistry of Curcumin: From Extraction to Therapeutic Agent. Molecules.

[10] He Y., Yue Y., Zheng X., Zhang K., Chen S., Du Z. (2015). Curcumin, Inflammation, and Chronic Diseases: How Are They Linked?. Molecules.

[11] Kumari P., Swami M.O., Nadipalli S.K., Myneni S., Ghosh B., Biswas S. (2016). Curcumin Delivery by Poly(Lactide)-Based Co-Polymeric Micelles: An In Vitro Anticancer Study. Pharm. Res..

[12] Matthewman C., Krishnakumar I.M., Swick A.G. (2024). Review: Bioavailability and Efficacy of “free” Curcuminoids from Curcumagalactomannoside (CGM) Curcumin Formulation. Nutr. Res. Rev..

[13] Jäger R., Lowery R.P., Calvanese A.V., Joy J.M., Purpura M., Wilson J.M. (2014). Comparative Absorption of Curcumin Formulations. Nutr. J..

[14] Stati G., Rossi F., Sancilio S., Basile M., Di Pietro R. (2021). *Curcuma longa* Hepatotoxicity: A Baseless Accusation. Cases Assessed for Causality Using RUCAM Method. Front. Pharmacol..

[15] Di Giacomo S., Briz O., Vitalone A., Di Sotto A. (2022). Editorial: Natural Products and Hepatic Health: Light and Shadows. Front. Pharmacol..

[16] Hussain Y., Abdullah, Khan F., Alsharif K.F., Alzahrani K.J., Saso L., Khan H. (2022). Regulatory Effects of Curcumin on Platelets: An Update and Future Directions. Biomedicines.

[17] Olas B. (2022). The Antioxidant, Anti-Platelet and Anti-Coagulant Properties of Phenolic Compounds, Associated with Modulation of Hemostasis and Cardiovascular Disease, and Their Possible Effect on COVID-19. Nutrients.

[18] Mashayekhi-Sardoo H., Mashayekhi-Sardoo A., Roufogalis B.D., Jamialahmadi T., Sahebkar A. (2021). Impact of Curcumin on Microsomal Enzyme Activities: Drug Interaction and Chemopreventive Studies. Curr. Med. Chem..

[19] Jin H., Qiao F., Wang Y., Xu Y., Shang Y. (2015). Curcumin Inhibits Cell Proliferation and Induces Apoptosis of Human Non-Small Cell Lung Cancer Cells through the Upregulation of MiR-192-5p and Suppression of PI3K/Akt Signaling Pathway. Oncol. Rep..

[20] Guo L.D., Chen X.J., Hu Y.H., Yu Z.J., Wang D., Liu J.Z. (2013). Curcumin Inhibits Proliferation and Induces Apoptosis of Human Colorectal Cancer Cells by Activating the Mitochondria Apoptotic Pathway. Phytother. Res..

[21] Li P., Pu S., Lin C., He L., Zhao H., Yang C., Guo Z., Xu S., Zhou Z. (2022). Curcumin Selectively Induces Colon Cancer Cell Apoptosis and S Cell Cycle Arrest by Regulates Rb/E2F/P53 Pathway. J. Mol. Struct..

[22] Lim T.G., Lee S.Y., Huang Z., Lim D.Y., Chen H., Jung S.K., Bode A.M., Lee K.W., Dong Z. (2014). Curcumin Suppresses Proliferation of Colon Cancer Cells by Targeting CDK2. Cancer Prev. Res..

[23] Hu A., Huang J.J., Zhang J.F., Dai W.J., Li R.L., Lu Z.Y., Duan J.L., Li J.P., Chen X.P., Fan J.P. (2017). Curcumin Induces G2/M Cell Cycle Arrest and Apoptosis of Head and Neck Squamous Cell Carcinoma in Vitro and in Vivo through ATM/Chk2/P53-Dependent Pathway. Oncotarget.

[24] Cheng C., Jiao J.T., Qian Y., Guo X.Y., Huang J., Dai M.C., Zhang L., Ding X.P., Zong D., Shao J.F. (2016). Curcumin Induces G2/M Arrest and Triggers Apoptosis via FoxO1 Signaling in U87 Human Glioma Cells. Mol. Med. Rep..

[25] Berrak Ö., Akkoç Y., Arisan E.D., Çoker-Gürkan A., Obakan-Yerlikaya P., Palavan-Ünsal N. (2016). The Inhibition of PI3K and NFκB Promoted Curcumin-Induced Cell Cycle Arrest at G2/M via Altering Polyamine Metabolism in Bcl-2 Overexpressing MCF-7 Breast Cancer Cells. Biomed. Pharmacother..

[26] Park M.-J., Kim E.-H., Park I.-C., Lee H.-C., Woo S.-H., Lee J.-Y., Hong Y.-J., Rhee C.H., Choi S.-H., Shim B.-S. (2002). Curcumin Inhibits Cell Cycle Progression of Immortalized Human Umbilical Vein Endothelial (ECV304) Cells by up-Regulating Cyclin-Dependent Kinase Inhibitor, P21WAF1/CIP1, P27KIP1 and P53. Int. J. Oncol..

[27] Mukhopadhyay A., Banerjee S., Stafford L.J., Xia C., Liu M., Aggarwal B.B. (2002). Curcumin-Induced Suppression of Cell Proliferation Correlates with down-Regulation of Cyclin D1 Expression and CDK4-Mediated Retinoblastoma Protein Phosphorylation. Oncogene.

[28] Srivastava R.K., Chen Q., Siddiqui I., Sarva K., Shankar S. (2007). Cell Cycle Linkage of Curcumin-Induced Cell Cycle Arrest and Apoptosis by Cyclin-Dependent Kinase Inhibitor P21/WAF1/CIP1 Linkage of Curcumin-Induced Cell Cycle Arrest and Apoptosis by Cyclin-Dependent Kinase Inhibitor P21/WAF1/CIP1. Cell Cycle.

[29] Laubach V., Kaufmann R., Bernd A., Kippenberger S., Zöller N. (2019). Extrinsic or Intrinsic Apoptosis by Curcumin and Light: Still a Mystery. Int. J. Mol. Sci..

[30] Zhu G.H., Dai H.P., Shen Q., Ji O., Zhang Q., Zhai Y.L. (2016). Curcumin Induces Apoptosis and Suppresses Invasion through MAPK and MMP Signaling in Human Monocytic Leukemia SHI-1 Cells. Pharm. Biol..

[31] Wahl H., Tan L., Griffith K., Choi M., Liu J.R. (2007). Curcumin Enhances Apo2L/TRAIL-Induced Apoptosis in Chemoresistant Ovarian Cancer Cells. Gynecol. Oncol..

[32] Shankar S., Srivastava R.K. (2007). Bax and Bak Genes Are Essential for Maximum Apoptotic Response by Curcumin, a Polyphenolic Compound and Cancer Chemopreventive Agent Derived from Turmeric, *Curcuma longa*. Carcinogenesis.

[33] Gogada R., Amadori M., Zhang H., Jones A., Verone A., Pitarresi J., Jandhyam S., Prabhu V., Black J.D., Chandra D. (2011). Curcumin Induces Apaf-1-Dependent, P21-Mediated Caspase Activation and Apoptosis. Cell Cycle.

[34] Shankar S., Chen Q., Sarva K., Siddiqui I., Srivastava R.K. (2007). Curcumin Enhances the Apoptosis-Inducing Potential of TRAIL in Prostate Cancer Cells: Molecular Mechanisms of Apoptosis, Migration and Angiogenesis. J. Mol. Signal..

[35] Wang J.B., Qi L.L., Zheng S.D., Wu T.X. (2009). Curcumin Induces Apoptosis through the Mitochondria-Mediated Apoptotic Pathway in HT-29 Cells. J. Zhejiang Univ. Sci. B.

[36] Woo J.H., Kim Y.H., Choi Y.J., Kim D.G., Lee K.S., Bae J.H., Min D.S., Chang J.S., Jeong Y.J., Lee Y.H. (2003). Molecular Mechanisms of Curcumin-Induced Cytotoxicity: Induction of Apoptosis through Generation of Reactive Oxygen Species, down-Regulation of Bcl-XL and IAP, the Release of Cytochrome c and Inhibition of Akt. Carcinogenesis.

[37] Sikora E., Bielak-Z Mijewska A., Magalska A., Piwocka K., Mosieniak G., Kalinowska M., Widlak P., Cymerman I.A., Bujnicki J.M. (2006). Curcumin Induces Caspase-3-Dependent Apoptotic Pathway but Inhibits DNA Fragmentation Factor 40/Caspase-Activated DNase Endonuclease in Human Jurkat Cells. Mol. Cancer Ther..

[38] Tan T.W., Tsai H.R., Lu H.F., Lin H.L., Tsou M.F., Lin Y.T., Tsai H.Y., Chen Y.F., Chung J.G. (2006). Curcumin-Induced Cell Cycle Arrest and Apoptosis in Human Acute Promyelocytic Leukemia HL-60 Cells via MMP Changes and Caspase-3 Activation. Anticancer Res..

[39] Bianchi G., Ravera S., Traverso C., Amaro A., Piaggio F., Emionite L., Bachetti T., Pfeffer U., Raffaghello L. (2018). Curcumin Induces a Fatal Energetic Impairment in Tumor Cells in Vitro and in Vivo by Inhibiting ATP-Synthase Activity. Carcinogenesis.

[40] Su C.C., Chen G.W., Lin J.G., Wu L.T., Chung J.G. (2006). Curcumin Inhibits Cell Migration of Human Colon Cancer Colo 205 Cells through the Inhibition of Nuclear Factor Kappa B/P65 and Down-Regulates Cyclooxygenase-2 and Matrix Metalloproteinase-2 Expressions. Anticancer Res..

[41] Ghasemi F., Shafiee M., Banikazemi Z., Hossein Pourhanifeh M., Khanbabaei H., Shamshirian A., Moghadam S.A., Arefnezhad R., Sahebkar A., Avan A. (2019). Curcumin Inhibits NF-KB and Wnt/β-Catenin Pathways in Cervical Cancer Cells. Pathol.-Res. Pract..

[42] Kim J.H., Gupta S.C., Park B., Yadav V.R., Aggarwal B.B. (2012). Turmeric (*Curcuma longa*) Inhibits Inflammatory Nuclear Factor (NF)-ΚB and NF-ΚB-Regulated Gene Products and Induces Death Receptors Leading to Suppressed Proliferation, Induced Chemosensitization, and Suppressed Osteoclastogenesis. Mol. Nutr. Food. Res..

[43] Bachmeier B.E., Nerlich A.G., Iancu C.M., Cilli M., Schleicher E., Vené R., Dell’Eva R., Jochum M., Albini A., Pfeffer U. (2007). The Chemopreventive Polyphenol Curcumin Prevents Hematogenous Breast Cancer Metastases in Immunodeficient Mice. Cell. Physiol. Biochem..

[44] Xia L., Tan S., Zhou Y., Lin J., Wang H., Oyang L., Tian Y., Liu L., Su M., Wang H. (2018). Role of the NFκB-Signaling Pathway in Cancer. Onco. Targets Ther..

[45] Park M.H., Hong J.T. (2016). Roles of NF-ΚB in Cancer and Inflammatory Diseases and Their Therapeutic Approaches. Cells.

[46] Tong W., Wang Q., Sun D., Suo J. (2016). Curcumin Suppresses Colon Cancer Cell Invasion via AMPK-Induced Inhibition of NF–ΚB, UPA Activator and MMP9. Oncol. Lett..

[47] Aggarwal S., Ichikawa H., Takada Y., Sandur S.K., Shishodia S., Aggarwal B.B. (2006). Curcumin (Diferuloylmethane) down-Regulates Expression of Cell Proliferation and Antiapoptotic and Metastatic Gene Products through Suppression of IkappaBalpha Kinase and Akt Activation. Mol. Pharmacol..

[48] Jobin C., Bradham C.A., Russo M.P., Juma B., Narula A.S., Brenner D.A., Sartor R.B. (1999). Curcumin Blocks Cytokine-Mediated NF-ΚB Activation and Proinflammatory Gene Expression by Inhibiting Inhibitory Factor I-ΚB Kinase Activity. J. Immunol..

[49] Giordano A., Tommonaro G. (2019). Curcumin and Cancer. Nutrients.

[50] Blasius R., Reuter S., Henry E., Dicato M., Diederich M. (2006). Curcumin Regulates Signal Transducer and Activator of Transcription (STAT) Expression in K562 Cells. Biochem. Pharmacol..

[51] Shishodia S. (2013). Molecular Mechanisms of Curcumin Action: Gene Expression. BioFactors.

[52] Zoi V., Kyritsis A.P., Galani V., Lazari D., Sioka C., Voulgaris S., Alexiou G.A. (2024). The Role of Curcumin in Cancer: A Focus on the PI3K/Akt Pathway. Cancers.

[53] Zhang Z., Yi P., Tu C., Zhan J., Jiang L., Zhang F. (2019). Curcumin Inhibits ERK/c-Jun Expressions and Phosphorylation against Endometrial Carcinoma. BioMed Res. Int..

[54] Borges G.A., Elias S.T., Amorim B., de Lima C.L., Coletta R.D., Castilho R.M., Squarize C.H., Guerra E.N.S. (2020). Curcumin Downregulates the PI3K-AKT-MTOR Pathway and Inhibits Growth and Progression in Head and Neck Cancer Cells. Phytother. Res..

[55] Yu S., Shen G., Tin O.K., Kim J.H., Kong A.N.T. (2008). Curcumin Inhibits Akt/MTOR Signaling through Protein Phosphatase-Dependent Mechanism. Mol. Cancer Ther..

[56] Farghadani R., Naidu R. (2021). Curcumin: Modulator of Key Molecular Signaling Pathways in Hormone-Independent Breast Cancer. Cancers.

[57] Dytrych P., Kejík Z., Hajduch J., Kaplánek R., Veselá K., Kučnirová K., Skaličková M., Venhauerová A., Hoskovec D., Martásek P. (2023). Therapeutic Potential and Limitations of Curcumin as Antimetastatic Agent. Biomed. Pharmacother..

[58] Davoodvandi A., Farshadi M., Zare N., Akhlagh S.A., Alipour Nosrani E., Mahjoubin-Tehran M., Kangari P., Sharafi S.M., Khan H., Aschner M. (2021). Antimetastatic Effects of Curcumin in Oral and Gastrointestinal Cancers. Front. Pharmacol..

[59] Aziz M.N.M., Rahim N.F.C., Hussin Y., Yeap S.K., Masarudin M.J., Mohamad N.E., Akhtar M.N., Osman M.A., Cheah Y.K., Alitheen N.B. (2021). Anti-Metastatic and Anti-Angiogenic Effects of Curcumin Analog DK1 on Human Osteosarcoma Cells In Vitro. Pharmaceuticals.

[60] Fu Z., Chen X., Guan S., Yan Y., Lin H., Hua Z.C. (2015). Curcumin Inhibits Angiogenesis and Improves Defective Hematopoiesis Induced by Tumor-Derived VEGF in Tumor Model through Modulating VEGF-VEGFR2 Signaling Pathway. Oncotarget.

[61] Giménez-Bastida J.A., Ávila-Gálvez M.Á., Carmena-Bargueño M., Pérez-Sánchez H., Espín J.C., González-Sarrías A. (2022). Physiologically Relevant Curcuminoids Inhibit Angiogenesis via VEGFR2 in Human Aortic Endothelial Cells. Food Chem. Toxicol..

[62] Liao H., Wang Z., Deng Z., Ren H., Li X. (2015). Curcumin Inhibits Lung Cancer Invasion and Metastasis by Attenuating GLUT1/MT1-MMP/MMP2 Pathway. Int. J. Clin. Exp. Med..

[63] Bachmeier B.E., Killian P.H., Melchart D. (2018). The Role of Curcumin in Prevention and Management of Metastatic Disease. Int. J. Mol. Sci..

[64] Jang B.Y., Shin M.K., Han D.H., Sung J.S. (2023). Curcumin Disrupts a Positive Feedback Loop between ADMSCs and Cancer Cells in the Breast Tumor Microenvironment via the CXCL12/CXCR4 Axis. Pharmaceutics.

[65] Ming T., Tao Q., Tang S., Zhao H., Yang H., Liu M., Ren S., Xu H. (2022). Curcumin: An Epigenetic Regulator and Its Application in Cancer. Biomed. Pharmacother..

[66] Teiten M.-H., Diederich M. (2013). Curcumin as a Regulator of Epigenetic Events. Mol. Nutr. Food Res..

[67] Shanmugam M.K., Arfuso F., Chia J., Sng G., Bishayee A. (2019). Epigenetic Effects of Curcumin in Cancer Prevention. Epigenetics Cancer Prevention.

[68] Boyanapalli S.S.S., Kong A.N.T. (2015). “Curcumin, the King of Spices”: Epigenetic Regulatory Mechanisms in the Prevention of Cancer, Neurological, and Inflammatory Diseases. Curr. Pharmacol. Rep..

[69] Liu Y., Sun Y., Guo Y., Shi X., Chen X., Feng W., Wu L.L., Zhang J., Yu S., Wang Y. (2023). An Overview: The Diversified Role of Mitochondria in Cancer Metabolism. Int. J. Biol. Sci..

[70] Ghosh P., Vidal C., Dey S., Zhang L. (2020). Mitochondria Targeting as an Effective Strategy for Cancer Therapy. Int. J. Mol. Sci..

[71] Lebleu V.S., O’Connell J.T., Gonzalez Herrera K.N., Wikman H., Pantel K., Haigis M.C., De Carvalho F.M., Damascena A., Domingos Chinen L.T., Rocha R.M. (2014). PGC-1α Mediates Mitochondrial Biogenesis and Oxidative Phosphorylation in Cancer Cells to Promote Metastasis. Nat. Cell Biol..

[72] Parlani M., Jorgez C., Friedl P. (2023). Plasticity of Cancer Invasion and Energy Metabolism. Trends. Cell Biol..

[73] Avolio R., Matassa D.S., Criscuolo D., Landriscina M., Esposito F. (2020). Modulation of Mitochondrial Metabolic Reprogramming and Oxidative Stress to Overcome Chemoresistance in Cancer. Biomolecules.

[74] Pendleton K.E., Wang K., Echeverria G.V. (2023). Rewiring of Mitochondrial Metabolism in Therapy-Resistant Cancers: Permanent and Plastic Adaptations. Front. Cell Dev. Biol..

[75] Cui H., Kong Y., Zhang H. (2012). Oxidative Stress, Mitochondrial Dysfunction, and Aging. J. Signal. Transduct..

[76] Cadenas E., Davies K.J.A. (2000). Mitochondrial Free Radical Generation, Oxidative Stress, and Aging. Free Radic. Biol. Med..

[77] Casanova A., Wevers A., Navarro-Ledesma S., Pruimboom L. (2023). Mitochondria: It Is All about Energy. Front. Physiol..

[78] Huang R., Chen H., Liang J., Li Y., Yang J., Luo C., Tang Y., Ding Y., Liu X., Yuan Q. (2021). Dual Role of Reactive Oxygen Species and Their Application in Cancer Therapy. J. Cancer.

[79] Arfin S., Jha N.K., Jha S.K., Kesari K.K., Ruokolainen J., Roychoudhury S., Rathi B., Kumar D. (2021). Oxidative Stress in Cancer Cell Metabolism. Antioxidants.

[80] Sathyabhama M., Priya Dharshini L.C., Karthikeyan A., Kalaiselvi S., Min T. (2022). The Credible Role of Curcumin in Oxidative Stress-Mediated Mitochondrial Dysfunction in Mammals. Biomolecules.

[81] Ravera S., Bertola N., Pasquale C., Bruno S., Benedicenti S., Ferrando S., Zekiy A., Arany P., Amaroli A. (2021). 808-Nm Photobiomodulation Affects the Viability of a Head and Neck Squamous Carcinoma Cellular Model, Acting on Energy Metabolism and Oxidative Stress Production. Biomedicines.

[82] Sekiya M., Chiba E., Satoh M., Yamakoshi H., Iwabuchi Y., Futai M., Nakanishi-Matsui M. (2014). Strong Inhibitory Effects of Curcumin and Its Demethoxy Analog on *Escherichia coli* ATP Synthase F1 Sector. Int. J. Biol. Macromol..

[83] Hu Y., Cheng L., Du S., Wang K., Liu S. (2024). Antioxidant Curcumin Induces Oxidative Stress to Kill Tumor Cells (Review). Oncol. Lett..

[84] Srinivasan K.R. (1953). A Chromatographic Study of the Curcuminoids in *Curcuma longa* L.. J. Pharm. Pharmacol..

[85] Appendino G., Allegrini P., de Combarieu E., Novicelli F., Ramaschi G., Sardone N. (2022). Shedding Light on Curcumin Stability. Fitoterapia.

[86] Roman B., Retajczyk M., Sałaciński Ł., Pełech R. (2020). Curcumin-Properties, Applications and Modification of Structure. Mini. Rev. Org. Chem..

[87] Slika L., Patra D. (2020). A Short Review on Chemical Properties, Stability and Nano-Technological Advances for Curcumin Delivery. Expert. Opin. Drug Deliv..

[88] Priyadarsini K.I. (2009). Photophysics, Photochemistry and Photobiology of Curcumin: Studies from Organic Solutions, Bio-Mimetics and Living Cells. J. Photochem. Photobiol. C-Photochem. Rev..

[89] Zhang H.A., Pratap-Singh A., Kitts D.D. (2023). Effect of Pulsed Light on Curcumin Chemical Stability and Antioxidant Capacity. PLoS ONE.

[90] Bernd A. (2014). Visible Light and/or UVA Offer a Strong Amplification of the Anti-Tumor Effect of Curcumin. Phytochem. Rev..

[91] Tønnesen H.H., Karlsen J., van Henegouwen G.B. (1986). Studies on Curcumin and Curcuminoids. VIII. Photochemical Stability of Curcumin. Z. Lebensm.-Unters. Forsch..

[92] Lee W.-H., Loo C.-Y., Bebawy M., Luk F., Mason R.S., Rohanizadeh R. (2013). Curcumin and Its Derivatives: Their Application in Neuropharmacology and Neuroscience in the 21st Century. Curr. NeuroPharmacol..

[93] Chatterjee P., Dutta S.S., Agarwal M., Dey S., Chakraborty T. (2024). UV-A-Induced Photoisomerization and Photodimerization of Curcumin: An Ion Mobility Mass Spectrometry Study. ACS Publ..

[94] Marazzi M., Francés-Monerris A., Mourer M., Pasc A., Monari A. (2020). Trans-to-Cis Photoisomerization of Cyclocurcumin in Different Environments Rationalized by Computational Photochemistry. Phys. Chem. Chem. Phys..

[95] Ansari M.J., Ahmad S., Kohli K., Ali J., Khar R.K. (2005). Stability-Indicating HPTLC Determination of Curcumin in Bulk Drug and Pharmaceutical Formulations. J. Pharm. Biomed. Anal..

[96] Ravera S., Pasquale C., Panfoli I., Bozzo M., Agas D., Bruno S., Hamblin M.R., Amaroli A. (2024). Assessing the Effects of Curcumin and 450 Nm Photodynamic Therapy on Oxidative Metabolism and Cell Cycle in Head and Neck Squamous Cell Carcinoma: An In Vitro Study. Cancers.

[97] Allison R.R., Moghissi K. (2013). Photodynamic Therapy (PDT): PDT Mechanisms. Clin. Endosc..

[98] Castano A.P., Mroz P., Hamblin M.R. (2006). Photodynamic Therapy and Anti-Tumour Immunity. Nat. Rev. Cancer.

[99] Ochsner M. (1997). Photophysical and Photobiological Processes in the Photodynamic Therapy of Tumours. J. Photochem. Photobiol. B..

[100] Castano A.P., Demidova T.N., Hamblin M.R. (2005). Mechanisms in Photodynamic Therapy: Part Two—Cellular Signaling, Cell Metabolism and Modes of Cell Death. Photodiagn. Photodyn. Ther..

[101] Ito T. (1978). Cellular and Subcellular Mechanisms of Photodynamic Action: The 1O2 Hypothesis as a Driving Force in Recent Research. Photochem. Photobiol..

[102] Rosenthal I., Ben-Hur E. (1995). Role of Oxygen in the Phototoxicity of Phthalocyanines. Int. J. Radiat. Biol..

[103] Sharma D., Singh S., Kumar P., Jain G.K., Aggarwal G., Almalki W.H., Kesharwani P. (2023). Mechanisms of Photodynamic Therapy. Nanomaterials for Photodynamic Therapy.

[104] Kah G., Chandran R., Abrahamse H. (2023). Curcumin a Natural Phenol and Its Therapeutic Role in Cancer and Photodynamic Therapy: A Review. Pharmaceutics.

[105] Dias L.D., Blanco K.C., Mfouo-Tynga I.S., Inada N.M., Bagnato V.S. (2020). Curcumin as a Photosensitizer: From Molecular Structure to Recent Advances in Antimicrobial Photodynamic Therapy. J. Photochem. Photobiol. C. Photochem. Rev..

[106] Zheng D., Huang C., Huang H., Zhao Y., Khan M.R.U., Zhao H., Huang L. (2020). Antibacterial Mechanism of Curcumin: A Review. Chem. Biodivers..

[107] Costa S.B., Campos A.C.C., Pereira A.C.M., de Mattos-Guaraldi A.L., Júnior R.H., Rosa A.C.P., Asad L.M.B.d.O. (2014). Adherence to Abiotic Surface Induces SOS Response in Escherichia Coli K-12 Strains under Aerobic and Anaerobic Conditions. Microbiology.

[108] Sharma G., Raturi K., Dang S., Gupta S., Gabrani R. (2014). Combinatorial Antimicrobial Effect of Curcumin with Selected Phytochemicals on Staphylococcus Epidermidis. J. Asian Nat. Prod. Res..

[109] Gayani B., Dilhari A., Wijesinghe G.K., Kumarage S., Abayaweera G., Samarakoon S.R., Perera I.C., Kottegoda N., Weerasekera M.M. (2019). Effect of Natural Curcuminoids-intercalated Layered Double Hydroxide Nanohybrid against *Staphylococcus aureus*, *Pseudomonas aeruginosa*, and *Enterococcus faecalis*: A Bactericidal, Antibiofilm, and Mechanistic Study. Microbiologyopen.

[110] Packiavathy I.A.S.V., Priya S., Pandian S.K., Ravi A.V. (2014). Inhibition of Biofilm Development of Uropathogens by Curcumin–An Anti-Quorum Sensing Agent from *Curcuma longa*. Food Chem..

[111] Izui S., Sekine S., Maeda K., Kuboniwa M., Takada A., Amano A., Nagata H. (2016). Antibacterial Activity of Curcumin Against Periodontopathic Bacteria. J. Periodontol..

[112] Pileggi G., Wataha J.C., Girard M., Grad I., Schrenzel J., Lange N., Bouillaguet S. (2013). Blue Light-Mediated Inactivation of Enterococcus Faecalis in Vitro. Photodiagn. Photodyn. Ther..

[113] Picco D.d.C.R., Cavalcante L.L.R., Trevisan R.L.B., Souza-Gabriel A.E., Borsatto M.C., Corona S.A.M. (2019). Effect of Curcumin-Mediated Photodynamic Therapy on Streptococcus Mutans and Candida Albicans: A Systematic Review of in Vitro Studies. Photodiagn. Photodyn. Ther..

[114] Pan H., Wang D., Zhang F. (2020). In Vitro Antimicrobial Effect of Curcumin-Based Photodynamic Therapy on *Porphyromonas gingivalis* and *Aggregatibacter actinomycetemcomitans*. Photodiagn. Photodyn. Ther..

[115] Pinto J.G., Fontana L.C., de Oliveira M.A., Kurachi C., Raniero L.J., Ferreira-Strixino J. (2016). In Vitro Evaluation of Photodynamic Therapy Using Curcumin on Leishmania Major and *Leishmania braziliensis*. Lasers. Med. Sci..

[116] Dong Q.L., Xing X.Y. (2018). Cancer Cells Arise from Bacteria. Cancer Cell Int..

[117] Lopez L.R., Bleich R.M., Arthur J.C. (2021). Microbiota Effects on Carcinogenesis: Initiation, Promotion, and Progression. Annu. Rev. Med..

[118] Sheweita S.A., Alsamghan A.S. (2020). Molecular Mechanisms Contributing Bacterial Infections to the Incidence of Various Types of Cancer. Mediat. Inflamm..

[119] Whitmore S.E., Lamont R.J. (2014). Oral Bacteria and Cancer. PLoS Pathog..

[120] Vega-Benedetti A.F., Loi E., Zavattari P. (2022). DNA Methylation Alterations Caused by Leishmania Infection May Generate a Microenvironment Prone to Tumour Development. Front. Cell Infect. Microbiol..

[121] Xie L., Ji X., Zhang Q., Wei Y. (2022). Curcumin Combined with Photodynamic Therapy, Promising Therapies for the Treatment of Cancer. Biomed. Pharmacother..

[122] Srivastava R.M., Singh S., Dubey S.K., Misra K., Khar A. (2011). Immunomodulatory and Therapeutic Activity of Curcumin. Int. ImmunoPharmacol..

[123] Machado F.C., Adum de Matos R.P., Primo F.L., Tedesco A.C., Rahal P., Calmon M.F. (2019). Effect of Curcumin-Nanoemulsion Associated with Photodynamic Therapy in Breast Adenocarcinoma Cell Line. Bioorg. Med. Chem..

[124] Sun M., Zhang Y., He Y., Xiong M., Huang H., Pei S., Liao J., Wang Y., Shao D. (2019). Green Synthesis of Carrier-Free Curcumin Nanodrugs for Light-Activated Breast Cancer Photodynamic Therapy. Colloids Surf. B Biointerfaces.

[125] Khorsandi K., Hosseinzadeh R., Shahidi F.K. (2019). Photodynamic Treatment with Anionic Nanoclays Containing Curcumin on Human Triple-Negative Breast Cancer Cells: Cellular and Biochemical Studies. J. Cell Biochem..

[126] Zhang J., Liang Y.C., Lin X., Zhu X., Yan L., Li S., Yang X., Zhu G., Rogach A.L., Yu P.K.N. (2015). Self-Monitoring and Self-Delivery of Photosensitizer-Doped Nanoparticles for Highly Effective Combination Cancer Therapy in Vitro and in Vivo. ACS Nano.

[127] Prathyusha E., A P., Ahmed H., Dethe M.R., Agrawal M., Gangipangi V., Sudhagar S., Krishna K.V., Dubey S.K., Pemmaraju D.B. (2022). Investigation of ROS Generating Capacity of Curcumin-Loaded Liposomes and Its in Vitro Cytotoxicity on MCF-7 Cell Lines Using Photodynamic Therapy. Photodiagn. Photodyn. Ther..

[128] Shao L., Zhu Y., Liao B., Wang G., Huang L., Yu L., Bai D. (2022). Effects of Curcumin-Mediated Photodynamic Therapy on Autophagy and Epithelial-Mesenchymal Transition of Lung Cancer Cells. Photodiagn. Photodyn. Ther..

[129] Bechnak L., Khalil C., El Kurdi R., Khnayzer R.S., Patra D. (2020). Curcumin Encapsulated Colloidal Amphiphilic Block Co-Polymeric Nanocapsules: Colloidal Nanocapsules Enhance Photodynamic and Anticancer Activities of Curcumin. Photochem. Photobiol. Sci..

[130] Szlasa W., Supplitt S., Drąg-Zalesińska M., Przystupski D., Kotowski K., Szewczyk A., Kasperkiewicz P., Saczko J., Kulbacka J. (2020). Effects of Curcumin Based PDT on the Viability and the Organization of Actin in Melanotic (A375) and Amelanotic Melanoma (C32)—in Vitro Studies. Biomed. Pharmacother..

[131] Woźniak M., Nowak M., Lazebna A., Więcek K., Jabłońska I., Szpadel K., Grzeszczak A., Gubernator J., Ziółkowski P. (2021). The Comparison of In Vitro Photosensitizing Efficacy of Curcumin-Loaded Liposomes Following Photodynamic Therapy on Melanoma MUG-Mel2, Squamous Cell Carcinoma SCC-25, and Normal Keratinocyte HaCaT Cells. Pharmaceuticals.

[132] Kazantzis K.T., Koutsonikoli K., Mavroidi B., Zachariadis M., Alexiou P., Pelecanou M., Politopoulos K., Alexandratou E., Sagnou M. (2020). Curcumin Derivatives as Photosensitizers in Photodynamic Therapy: Photophysical Properties and in Vitro Studies with Prostate Cancer Cells. Photochem. Photobiol. Sci..

[133] He G., Mu T., Yuan Y., Yang W., Zhang Y., Chen Q., Bian M., Pan Y., Xiang Q., Chen Z. (2019). Effects of Notch Signaling Pathway in Cervical Cancer by Curcumin Mediated Photodynamic Therapy and Its Possible Mechanisms in Vitro and in Vivo. J. Cancer.

[134] Lin Y.H., Chen C.Y. (2020). Folate-Targeted Curcumin-Encapsulated Micellar Nanosystem for Chemotherapy and Curcumin-Mediated Photodynamic Therapy. Polymers.

[135] Jamali Z., Khoobi M., Hejazi S.M., Eivazi N., Abdolahpour S., Imanparast F., Moradi-Sardareh H., Paknejad M. (2018). Evaluation of Targeted Curcumin (CUR) Loaded PLGA Nanoparticles for in Vitro Photodynamic Therapy on Human Glioblastoma Cell Line. Photodiagn. Photodyn. Ther..

[136] Beyer K., Nikfarjam F., Butting M., Meissner M., König A., Bosca A.R., Kaufmann R., Heidemann D., Bernd A., Kippenberger S. (2017). Photodynamic Treatment of Oral Squamous Cell Carcinoma Cells with Low Curcumin Concentrations. J. Cancer.

[137] Dujic J., Kippenberger S., Ramirez-Bosca A., Diaz-Alperi J., Bereiter-Hahn J., Kaufmann R., Bernd A., Hofmann M. (2009). Curcumin in Combination with Visible Light Inhibits Tumor Growth in a Xenograft Tumor Model. Int. J. Cancer.

[138] Tønnesen H.H., Másson M., Loftsson T. (2002). Studies of Curcumin and Curcuminoids. XXVII. Cyclodextrin Complexation: Solubility, Chemical and Photochemical Stability. Int. J. Pharm..

[139] Pan-On S., Dilokthornsakul P., Tiyaboonchai W. (2022). Trends in Advanced Oral Drug Delivery System for Curcumin: A Systematic Review. J. Control Release.

[140] Mirzaie Z., Barati M., Tokmedash M.A. (2020). Anticancer Drug Delivery Systems Based on Curcumin Nanostructures: A Review. Pharm. Chem. J.

[141] Mirzaei H., Shakeri A., Rashidi B., Jalili A., Banikazemi Z., Sahebkar A. (2017). Phytosomal Curcumin: A Review of Pharmacokinetic, Experimental and Clinical Studies. Biomed. Pharmacother..

[142] Purpura M., Lowery R.P., Wilson J.M., Mannan H., Münch G., Razmovski-Naumovski V. (2018). Analysis of Different Innovative Formulations of Curcumin for Improved Relative Oral Bioavailability in Human Subjects. Eur. J. Nutr..

[143] Munjal B., Pawar Y.B., Patel S.B., Bansal A.K. (2011). Comparative Oral Bioavailability Advantage from Curcumin Formulations. Drug Deliv. Transl. Res..

[144] Belcaro G., Hosoi M., Pellegrini L., Appendino G., Ippolito E., Ricci A., Ledda A., Dugall M., Cesarone M.R., Maione C. (2014). A Controlled Study of a Lecithinized Delivery System of Curcumin (Meriva®) to Alleviate the Adverse Effects of Cancer Treatment. Phytother. Res..

[145] Ledda A., Belcaro G., Dugall M., Luzzi R., Scoccianti M., Togni S., Appendino G., Ciammaichella G. (2012). Meriva®, a lecithinized curcumin delivery system, in the control of benign prostatic hyperplasia: A pilot, product evaluation registry study. Panminerva Medica.

[146] Panahi Y., Alishiri G.H., Parvin S., Sahebkar A. (2016). Mitigation of Systemic Oxidative Stress by Curcuminoids in Osteoarthritis: Results of a Randomized Controlled Trial. J. Diet Suppl..

[147] Barhoumi R., Ibrahim A., El-Meligy A., Fetaih H., Dessouki A., Stoica G. (2010). Effect of Curcumin and Meriva on the Lung Metastasis of Murine Mammary Gland Adenocarcinoma. In Vivo.

[148] Gong J., Chen M., Zheng Y., Wang S., Wang Y. (2012). Polymeric Micelles Drug Delivery System in Oncology. J. Control. Release.

[149] Flory S., Sus N., Haas K., Jehle S., Kienhöfer E., Waehler R., Adler G., Venturelli S., Frank J. (2021). Increasing Post-Digestive Solubility of Curcumin Is the Most Successful Strategy to Improve Its Oral Bioavailability: A Randomized Cross-Over Trial in Healthy Adults and In Vitro Bioaccessibility Experiments. Mol. Nutr. Food Res..

[150] Khalil N.M., do Nascimento T.C.F., Casa D.M., Dalmolin L.F., de Mattos A.C., Hoss I., Romano M.A., Mainardes R.M. (2013). Pharmacokinetics of Curcumin-Loaded PLGA and PLGA–PEG Blend Nanoparticles after Oral Administration in Rats. Colloids Surf. B Biointerfaces.

[151] Duan Y., Zhang B., Chu L., Tong H.H.Y., Liu W., Zhai G. (2016). Evaluation in Vitro and in Vivo of Curcumin-Loaded MPEG-PLA/TPGS Mixed Micelles for Oral Administration. Colloids Surf. B. Biointerfaces.

[152] Khatik R., Mishra R., Verma A., Dwivedi P., Kumar V., Gupta V., Paliwal S.K., Mishra P.R., Dwivedi A.K. (2013). Colon-Specific Delivery of Curcumin by Exploiting Eudragit-Decorated Chitosan Nanoparticles in Vitro and in Vivo. J. Nanopart. Res..

[153] Xie X., Tao Q., Zou Y., Zhang F., Guo M., Wang Y., Wang H., Zhou Q., Yu S. (2011). PLGA Nanoparticles Improve the Oral Bioavailability of Curcumin in Rats: Characterizations and Mechanisms. J. Agric. Food Chem..

[154] Esfandiarpour-Boroujeni S., Bagheri-Khoulenjani S., Mirzadeh H., Amanpour S. (2017). Fabrication and Study of Curcumin Loaded Nanoparticles Based on Folate-Chitosan for Breast Cancer Therapy Application. Carbohydr. Polym..

[155] Muddineti O.S., Kumari P., Ghosh B., Torchilin V.P., Biswas S. (2017). D-α-Tocopheryl Succinate/Phosphatidyl Ethanolamine Conjugated Amphiphilic Polymer-Based Nanomicellar System for the Efficient Delivery of Curcumin and To Overcome Multiple Drug Resistance in Cancer. ACS Appl. Mater. Interfaces.

[156] Abruzzo A., Zuccheri G., Belluti F., Provenzano S., Verardi L., Bigucci F., Cerchiara T., Luppi B., Calonghi N. (2016). Chitosan Nanoparticles for Lipophilic Anticancer Drug Delivery: Development, Characterization and in Vitro Studies on HT29 Cancer Cells. Colloids Surf. B Biointerfaces.

[157] Yallapu M.M., Jaggi M., Chauhan S.C. (2010). Beta-Cyclodextrin-Curcumin Self-Assembly Enhances Curcumin Delivery in Prostate Cancer Cells. Colloids Surf. B Biointerfaces.

[158] Shahriari M., Kesharwani P., Johnston T.P., Sahebkar A. (2023). Anticancer Potential of Curcumin-Cyclodextrin Complexes and Their Pharmacokinetic Properties. Int. J. Pharm..

[159] Samad A., Sultana Y., Aqil M. (2007). Liposomal Drug Delivery Systems: An Update Review. Curr. Drug Deliv..

[160] Li R., Deng L., Cai Z., Zhang S., Wang K., Li L., Ding S., Zhou C. (2017). Liposomes Coated with Thiolated Chitosan as Drug Carriers of Curcumin. Mater. Sci. Eng. C Mater. Biol. Appl..

[161] Cuomo F., Cofelice M., Venditti F., Ceglie A., Miguel M., Lindman B., Lopez F. (2018). In-Vitro Digestion of Curcumin Loaded Chitosan-Coated Liposomes. Colloids Surf. B Biointerfaces.

[162] Takahashi M., Uechi S., Takara K., Asikin Y., Wada K. (2009). Evaluation of an Oral Carrier System in Rats: Bioavailability and Antioxidant Properties of Liposome-Encapsulated Curcumin. J. Agric. Food Chem..

[163] Chen H., Wu J., Sun M., Guo C., Yu A., Cao F., Zhao L., Tan Q., Zhai G. (2012). N-Trimethyl Chitosan Chloride-Coated Liposomes for the Oral Delivery of Curcumin. J. Liposome Res..

[164] Ng Z.Y., Wong J.Y., Panneerselvam J., Madheswaran T., Kumar P., Pillay V., Hsu A., Hansbro N., Bebawy M., Wark P. (2018). Assessing the Potential of Liposomes Loaded with Curcumin as a Therapeutic Intervention in Asthma. Colloids Surf. B Biointerfaces.

[165] Wang L.Q., Shi H.S., Wang Y.S. (2013). Liposomal Curcumin Inhibits Tumor Growth and Angiogenesis in Lewis Lung Cancer. J. Sichuan Univ. (Med. Sci.).

[166] Lin Y.L., Liu Y.K., Tsai N.M., Hsieh J.H., Chen C.H., Lin C.M., Liao K.W. (2012). A Lipo-PEG-PEI Complex for Encapsulating Curcumin That Enhances Its Antitumor Effects on Curcumin-Sensitive and Curcumin-Resistance Cells. Nanomedicine.

[167] Rahman S., Cao S., Steadman K.J., Wei M., Parekh H.S. (2012). Native and β-Cyclodextrin-Enclosed Curcumin: Entrapment within Liposomes and Their in Vitro Cytotoxicity in Lung and Colon Cancer. Drug Deliv..

[168] Saengkrit N., Saesoo S., Srinuanchai W., Phunpee S., Ruktanonchai U.R. (2014). Influence of Curcumin-Loaded Cationic Liposome on Anticancer Activity for Cervical Cancer Therapy. Colloids Surf. B Biointerfaces.

[169] Huang Q., Zhang L., Sun X., Zeng K., Li J., Liu Y.N. (2014). Coating of Carboxymethyl Dextran on Liposomal Curcumin to Improve the Anticancer Activity. RSC Adv..

[170] Tian Y., Guan Y.B., Zhang Y.Q., Wei X.C., Du Z.Y., Conney A.H., Zheng X. (2014). Inhibitory Effect of Curcumin Liposomes on PC-3 Human Prostate Cancer Cells. Chin. J. Exp. Surg..

[171] Ibrahim S., Tagami T., Kishi T., Ozeki T. (2018). Curcumin Marinosomes as Promising Nano-Drug Delivery System for Lung Cancer. Int. J. Pharm..

[172] Hasan M., Belhaj N., Benachour H., Barberi-Heyob M., Kahn C.J.F., Jabbari E., Linder M., Arab-Tehrany E. (2014). Liposome Encapsulation of Curcumin: Physico-Chemical Characterizations and Effects on MCF7 Cancer Cell Proliferation. Int. J. Pharm..

[173] Dhule S.S., Penfornis P., Frazier T., Walker R., Feldman J., Tan G., He J., Alb A., John V., Pochampally R. (2012). Curcumin-Loaded γ-Cyclodextrin Liposomal Nanoparticles as Delivery Vehicles for Osteosarcoma. Nanomedicine.

[174] Koksharov Y.A., Gubin S.P., Taranov I.V., Khomutov G.B., Gulyaev Y.V. (2022). Magnetic Nanoparticles in Medicine: Progress, Problems, and Advances. J. Commun. Technol. Electron..

[175] Indira T.K., Lakshmi P.K. (2010). Magnetic Nanoparticles—A Review. Int. J. Pharm. Sci. Nanotechnol. (IJPSN).

[176] Rezaei B., Yari P., Sanders S.M., Wang H., Chugh V.K., Liang S., Mostufa S., Xu K., Wang J.P., Gómez-Pastora J. (2024). Magnetic Nanoparticles: A Review on Synthesis, Characterization, Functionalization, and Biomedical Applications. Small.

[177] Nosrati H., Charmi J., Abedini S., Rashidi N., Attari E., Davaran S., Danafar H., Kheiri Manjili H. (2018). Preparation and Characterization of Magnetic Theranostic Nanoparticles for Curcumin Delivery and Evaluation as MRI Contrast Agent. Appl. Organomet. Chem..

[178] Patil P.B., Parit S.B., Waifalkar P.P., Patil S.P., Dongale T.D., Sahoo S.C., Kollu P., Nimbalkar M.S., Patil P.S., Chougale A.D. (2018). PH Triggered Curcumin Release and Antioxidant Activity of Curcumin Loaded γ-Fe_2_O_3_ Magnetic Nanoparticles. Mater. Lett..

[179] Cui Y., Zhang M., Zeng F., Jin H., Xu Q., Huang Y. (2016). Dual-Targeting Magnetic PLGA Nanoparticles for Codelivery of Paclitaxel and Curcumin for Brain Tumor Therapy. ACS Appl. Mater. Interfaces.

[180] Ramezani Farani M., Azarian M., Heydari Sheikh Hossein H., Abdolvahabi Z., Mohammadi Abgarmi Z., Moradi A., Mousavi S.M., Ashrafizadeh M., Makvandi P., Saeb M.R. (2022). Folic Acid-Adorned Curcumin-Loaded Iron Oxide Nanoparticles for Cervical Cancer. ACS Appl. Bio Mater..

[181] Pazouki N., Irani S., Olov N., Atyabi S.M., Bagheri-Khoulenjani S. (2022). Fe_3_O_4_ Nanoparticles Coated with Carboxymethyl Chitosan Containing Curcumin in Combination with Hyperthermia Induced Apoptosis in Breast Cancer Cells. Prog. Biomater..

[182] Bourang S., Asadian S., Noruzpour M., Mansuryar A., Azizi S., Ebrahimi H.A., Amani Hooshyar V. (2024). PLA-HA/Fe_3_O_4_ Magnetic Nanoparticles Loaded with Curcumin: Physicochemical Characterization and Toxicity Evaluation in HCT116 Colorectal Cancer Cells. Discov. Appl. Sci..

[183] Fereydouni N., Darroudi M., Movaffagh J., Shahroodi A., Butler A.E., Ganjali S., Sahebkar A. (2019). Curcumin Nanofibers for the Purpose of Wound Healing. J. Cell Physiol..

[184] Thangaraju E., Srinivasan N.T., Kumar R., Sehgal P.K., Rajiv S. (2012). Fabrication of Electrospun Poly L-Lactide and Curcumin Loaded Poly L-Lactide Nanofibers for Drug Delivery. Fibers Polym..

[185] Mohebian Z., Babazadeh M., Zarghami N., Mousazadeh H. (2021). Anticancer Efficiency of Curcumin-Loaded Mesoporous Silica Nanoparticles/Nanofiber Composites for Potential Postsurgical Breast Cancer Treatment. J. Drug Deliv. Sci. Technol..

[186] Nguyen T.T.T., Ghosh C., Hwang S.G., Tran L.D., Park J.S. (2013). Characteristics of Curcumin-Loaded Poly (Lactic Acid) Nanofibers for Wound Healing. J. Mater. Sci..

[187] Akrami-Hasan-Kohal M., Tayebi L., Ghorbani M. (2020). Curcumin-Loaded Naturally-Based Nanofibers as Active Wound Dressing Mats: Morphology, Drug Release, Cell Proliferation, and Cell Adhesion Studies. New J. Chem..

[188] Elakkiya T., Malarvizhi G., Rajiv S., Natarajan T.S. (2014). Curcumin Loaded Electrospun Bombyx Mori Silk Nanofibers for Drug Delivery. Polym. Int..

[189] Rezaei A., Nasirpour A. (2019). Evaluation of Release Kinetics and Mechanisms of Curcumin and Curcumin-β-Cyclodextrin Inclusion Complex Incorporated in Electrospun Almond Gum/PVA Nanofibers in Simulated Saliva and Simulated Gastrointestinal Conditions. Bionanoscience.

[190] Cheng T., Zhang Z., Shen H., Jian Z., Li J., Chen Y., Shen Y., Dai X. (2020). Topically Applicated Curcumin/Gelatin-Blended Nanofibrous Mat Inhibits Pancreatic Adenocarcinoma by Increasing ROS Production and Endoplasmic Reticulum Stress Mediated Apoptosis. J. Nanobiotechnol..

[191] Razmshoar P., Bahrami S.H., Akbari S. (2020). Functional Hydrophilic Highly Biodegradable PCL Nanofibers through Direct Aminolysis of PAMAM Dendrimer. Int. J. Polym. Mater. Polym. Biomater..

[192] Sedghi R., Shaabani A., Mohammadi Z., Samadi F.Y., Isaei E. (2017). Biocompatible Electrospinning Chitosan Nanofibers: A Novel Delivery System with Superior Local Cancer Therapy. Carbohydr. Polym..

[193] Guo F., Guo D., Zhang W., Yan Q., Yang Y., Hong W., Yang G. (2017). Preparation of Curcumin-Loaded PCL-PEG-PCL Triblock Copolymeric Nanoparticles by a Microchannel Technology. Eur. J. Pharm. Sci..

[194] Anuchapreeda S., Fukumori Y., Okonogi S., Ichikawa H. (2012). Preparation of Lipid Nanoemulsions Incorporating Curcumin for Cancer Therapy. J. Nanotechnol..

[195] Jiang T., Liao W., Charcosset C. (2020). Recent Advances in Encapsulation of Curcumin in Nanoemulsions: A Review of Encapsulation Technologies, Bioaccessibility and Applications. Food Res. Int..

[196] Cuomo F., Perugini L., Marconi E., Messia M.C., Lopez F. (2019). Enhanced Curcumin Bioavailability through Nonionic Surfactant/Caseinate Mixed Nanoemulsions. J. Food Sci..

[197] Xu G., Wang C., Yao P. (2017). Stable Emulsion Produced from Casein and Soy Polysaccharide Compacted Complex for Protection and Oral Delivery of Curcumin. Food Hydrocoll..

[198] Peng S., Li Z., Zou L., Liu W., Liu C., McClements D.J. (2018). Enhancement of Curcumin Bioavailability by Encapsulation in Sophorolipid-Coated Nanoparticles: An in Vitro and in Vivo Study. J. Agric. Food Chem..

[199] Hu L., Jia Y., Niu F., Jia Z., Yang X., Jiao K. (2012). Preparation and Enhancement of Oral Bioavailability of Curcumin Using Microemulsions Vehicle. J. Agric. Food Chem..

[200] Ochoa-Flores A.A., Hernández-Becerra J.A., Cavazos-Garduño A., Soto-Rodríguez I., Guadalupe Sanchez-Otero M., Vernon-Carter E.J., García H.S. (2016). Enhanced Bioavailability of Curcumin Nanoemulsions Stabilized with Phosphatidylcholine Modified with Medium Chain Fatty Acids. Curr. Drug Deliv..

[201] Onoue S., Takahashi H., Kawabata Y., Seto Y., Hatanaka J., Timmermann B., Yamada S. (2010). Formulation Design and Photochemical Studies on Nanocrystal Solid Dispersion of Curcumin with Improved Oral Bioavailability. J. Pharm. Sci..

[202] Boscán F., Barandiaran M.J., Paulis M. (2018). From Miniemulsion to Nanoemulsion Polymerization of Superhydrophobic Monomers through Low Energy Phase Inversion Temperature. J. Ind. Eng. Chem..

[203] Calderó G., Montes R., Llinàs M., García-Celma M.J., Porras M., Solans C. (2016). Studies on the Formation of Polymeric Nano-Emulsions Obtained via Low-Energy Emulsification and Their Use as Templates for Drug Delivery Nanoparticle Dispersions. Colloids Surf. B Biointerfaces.

[204] Chen Y.C., Chen B.H. (2018). Preparation of Curcuminoid Microemulsions from *Curcuma longa* L. to Enhance Inhibition Effects on Growth of Colon Cancer Cells HT-29. RSC Adv..

[205] Peng Y., Yu S., Wang Z., Huang P., Wang W., Xing J. (2022). Nanogels Loading Curcumin in Situ through Microemulsion Photopolymerization for Enhancement of Antitumor Effects. J. Mater. Chem. B.

[206] Guerrero S., Inostroza-Riquelme M., Contreras-Orellana P., Diaz-Garcia V., Lara P., Vivanco-Palma A., Cárdenas A., Miranda V., Robert P., Leyton L. (2018). Curcumin-Loaded Nanoemulsion: A New Safe and Effective Formulation to Prevent Tumor Reincidence and Metastasis. Nanoscale.

[207] Notarbartolo M., Poma P., Perri D., Dusonchet L., Cervello M., D’Alessandro N. (2005). Antitumor Effects of Curcumin, Alone or in Combination with Cisplatin or Doxorubicin, on Human Hepatic Cancer Cells. Analysis of Their Possible Relationship to Changes in NF-KB Activation Levels and in IAP Gene Expression. Cancer Lett..

[208] Ombredane A.S., Silva V.R.P., Andrade L.R., Pinheiro W.O., Simonelly M., Oliveira J.V., Pinheiro A.C., Gonçalves G.F., Felice G.J., Garcia M.P. (2021). In Vivo Efficacy and Toxicity of Curcumin Nanoparticles in Breast Cancer Treatment: A Systematic Review. Front. Oncol..

[209] Sethiya A., Agarwal D.K., Agarwal S. (2020). Current Trends in Drug Delivery System of Curcumin and Its Therapeutic Applications. Mini-Rev. Med. Chem..

[210] Zhang M., Zhuang B., Du G., Han G., Jin Y. (2019). Curcumin Solid Dispersion-Loaded in Situ Hydrogels for Local Treatment of Injured Vaginal Bacterial Infection and Improvement of Vaginal Wound Healing. J. Pharm. Pharmacol..

[211] Rezvan G., Pircheraghi G., Bagheri R. (2018). Curcumin Incorporated PVA-Borax Dual Delivery Hydrogels as Potential Wound Dressing Materials—Correlation between Viscoelastic Properties and Curcumin Release Rate. J. Appl. Polym. Sci..

[212] Zheng B., Zhang Z., Chen F., Luo X., McClements D.J. (2017). Impact of Delivery System Type on Curcumin Stability: Comparison of Curcumin Degradation in Aqueous Solutions, Emulsions, and Hydrogel Beads. Food Hydrocoll..

[213] Stachowiak M., Mlynarczyk D.T., Dlugaszewska J. (2024). Wondrous Yellow Molecule: Are Hydrogels a Successful Strategy to Overcome the Limitations of Curcumin?. Molecules.

[214] Hussein Y., Loutfy S.A., Kamoun E.A., El-Moslamy S.H., Radwan E.M., Elbehairi S.E.I. (2021). Enhanced Anti-Cancer Activity by Localized Delivery of Curcumin Form PVA/CNCs Hydrogel Membranes: Preparation and in Vitro Bioevaluation. Int. J. Biol. Macromol..

[215] Sharma A.K., Kapoor V.K., Kaur G. (2022). Herb-Drug Interactions: A Mechanistic Approach. Drug Chem. Toxicol..

[216] Bahramsoltani R., Rahimi R., Farzaei M.H. (2017). Pharmacokinetic Interactions of Curcuminoids with Conventional Drugs: A Review. J. EthnoPharmacol..

[217] Choi J.G., Eom S.M., Kim J., Kim S.H., Huh E., Kim H., Lee Y., Lee H., Oh M.S. (2016). A Comprehensive Review of Recent Studies on Herb-Drug Interaction: A Focus on Pharmacodynamic Interaction. J. Altern. Complement. Med..

[218] Wang X. (2015). long Potential Herb-Drug Interaction in the Prevention of Cardiovascular Diseases during Integrated Traditional and Western Medicine Treatment. Chin. J. Integr. Med..

[219] Fugh-Berman A. (2000). Herb-Drug Interactions. Lancet.

[220] Rahimi R., Abdollahi M. (2012). An Update on the Ability of St. John’s Wort to Affect the Metabolism of Other Drugs. Expert Opin. Drug Metab. Toxicol..

[221] Appiah-Opong R., Commandeur J.N.M., van Vugt-Lussenburg B., Vermeulen N.P.E. (2007). Inhibition of Human Recombinant Cytochrome P450s by Curcumin and Curcumin Decomposition Products. Toxicology.

[222] Anuchapreeda S., Leechanachai P., Smith M.M., Ambudkar S.V., Limtrakul P. (2002). Modulation of P-Glycoprotein Expression and Function by Curcumin in Multidrug-Resistant Human KB Cells. Biochem. Pharmacol..

[223] Chearwae W., Wu C.P., Chu H.Y., Lee T.R., Ambudkar S.V., Limtrakul P. (2006). Curcuminoids Purified from Turmeric Powder Modulate the Function of Human Multidrug Resistance Protein 1 (ABCC1). Cancer Chemother. Pharmacol..

[224] Basu N.K., Kole L., Kubota S., Owens I.S. (2004). Human UDP-Glucuronosyltransferases Show Atypical Metabolism of Mycophenolic Acid and Inhibition by Curcumin. Drug Metab. Dispos..

[225] Volak L.P., Hanley M.J., Masse G., Hazarika S., Harmatz J.S., Badmaev V., Majeed M., Greenblatt D.J., Court M.H. (2013). Effect of a Herbal Extract Containing Curcumin and Piperine on Midazolam, Flurbiprofen and Paracetamol (Acetaminophen) Pharmacokinetics in Healthy Volunteers. Br. J. Clin. Pharmacol..

[226] Ganta S., Devalapally H., Amiji M. (2010). Curcumin Enhances Oral Bioavailability and Anti-Tumor Therapeutic Efficacy of Paclitaxel upon Administration in Nanoemulsion Formulation. J. Pharm. Sci..

[227] Sun X., Li J., Guo C., Xing H., Xu J., Wen Y., Qiu Z., Zhang Q., Zheng Y., Chen X. (2016). Pharmacokinetic Effects of Curcumin on Docetaxel Mediated by OATP1B1, OATP1B3 and CYP450s. Drug Metab. Pharmacokinet..

[228] Lee C.K., Ki S.H., Choi J.S. (2011). Effects of Oral Curcumin on the Pharmacokinetics of Intravenous and Oral Etoposide in Rats: Possible Role of Intestinal CYP3A and P-Gp Inhibition by Curcumin. Biopharm. Drug Dispos..

[229] Cho Y.A., Lee W., Choi J.S. (2012). Effects of Curcumin on the Pharmacokinetics of Tamoxifen and Its Active Metabolite, 4-Hydroxytamoxifen, in Rats: Possible Role of CYP3A4 and P-Glycoprotein Inhibition by Curcumin. Pharmazie.

[230] Cheng K.W., Wong C.C., Mattheolabakis G., Xie G., Huang L., Rigas B. (2013). Curcumin Enhances the Lung Cancer Chemopreventive Efficacy of Phospho-Sulindac by Improving Its Pharmacokinetics. Int. J. Oncol..

[231] Hsieh Y.W., Huang C.Y., Yang S.Y., Peng Y.H., Yu C.P., Chao P.D.L., Hou Y.C. (2014). Oral Intake of Curcumin Markedly Activated CYP 3A4: In Vivo and Ex-Vivo Studies. Sci. Rep..

[232] Zhang W., Tan T.M.C., Lim L.Y. (2007). Impact of Curcumin-Induced Changes in P-Glycoprotein and CYP3A Expression on the Pharmacokinetics of Peroral Celiprolol and Midazolam in Rats. Drug Metab. Dispos..

[233] Murad H.A.S., Suliaman M.I., Abdallah H., Abdulsattar M. (2014). Does Curcumin or Pindolol Potentiate Fluoxetine’s Antidepressant Effect by a Pharmacokinetic or Pharmacodynamic Interaction?. Indian J. Pharm. Sci..

[234] Kim S.-B., Cho S.-S., Cho H.-J., Yoon I.-S. (2015). Modulation of Hepatic Cytochrome P450 Enzymes by Curcumin and Its Pharmacokinetic Consequences in Sprague-Dawley Rats. Pharmacogn. Mag..

[235] Liu A.C., Zhao L.X., Xing J., Liu T., Du F.Y., Lou H.X. (2012). Pre-Treatment with Curcumin Enhances Plasma Concentrations of Losartan and Its Metabolite EXP3174 in Rats. Biol. Pharm. Bull..

[236] Zhou X., Zhang F., Chen C., Guo Z., Liu J., Yu J., Xu Y., Zhong D., Jiang H. (2017). Impact of Curcumin on the Pharmacokinetics of Rosuvastatin in Rats and Dogs Based on the Conjugated Metabolites. Xenobiotica.

[237] Juan H., Jing T., Wan-Hua Y., Juan S., Xiao-Lei L., Wen-Xing P. (2013). P-Gp Induction by Curcumin: An Effective Antidotal Pathway. J Bioequiv. Availab..

[238] Koonrungsesomboon N., Teekachunhatean S., Potikanond S., Hanprasertpong N. (2021). Unusual Pharmacokinetic Herb-Drug Interactions between Turmeric Crude Extract and Digoxin in Male Volunteers. J. Basic Appl. Pharmacol..

[239] Ikehata M., Ohnishi N., Egami S., Kishi H., Shin Y., Takara K., Tsuchishita Y., Tokuda N., Hori S., Yatani Y. (2008). Effects of Turmeric Extract on the Pharmacokinetics of Nifedipine After a Single Oral Administration in Healthy Volunteers. J. Diet Suppl..

[240] Liu A.C., Zhao L.X., Lou H.X. (2013). Curcumin Alters the Pharmacokinetics of Warfarin and Clopidogrel in Wistar Rats but Has No Effect on Anticoagulation or Antiplatelet Aggregation. Planta Med..

[241] Volak L.P., Ghirmai S., Cashman J.R., Court M.H. (2008). Curcuminoids Inhibit Multiple Human Cytochromes P450, UDP-Glucuronosyltransferase, and Sulfotransferase Enzymes, Whereas Piperine Is a Relatively Selective CYP3A4 Inhibitor. Drug Metab. Dispos..

[242] Pavithra B.H., Prakash N., Jayakumar K. (2009). Modification of Pharmacokinetics of Norfloxacin Following Oral Administration of Curcumin in Rabbits. J. Vet. Sci..

[243] Zhang D.W., Fu M., Gao S.H., Liu J.L. (2013). Curcumin and Diabetes: A Systematic Review. Evid.-Based Complement. Altern. Med..

[244] Sosa V., Moliné T., Somoza R., Paciucci R., Kondoh H., LLeonart M.E. (2013). Oxidative Stress and Cancer: An Overview. Ageing Res. Rev..

[245] Valko M., Leibfritz D., Moncol J., Cronin M.T.D., Mazur M., Telser J. (2007). Free Radicals and Antioxidants in Normal Physiological Functions and Human Disease. Int. J. Biochem. Cell Biol..

[246] Karin M. (2006). Nuclear Factor-KappaB in Cancer Development and Progression. Nature.

[247] Didonato J.A., Mercurio F., Karin M. (2012). NF-ΚB and the Link between Inflammation and Cancer. Immunol. Rev..

[248] Grivennikov S.I., Karin M. (2010). Dangerous Liaisons: STAT3 and NF-ΚB Collaboration and Crosstalk in Cancer. Cytokine Growth Factor Rev..

[249] Yu H., Pardoll D., Jove R. (2009). STATs in Cancer Inflammation and Immunity: A Leading Role for STAT3. Nat. Rev. Cancer.

[250] Cao W., Zhang Y., Li A., Yu P., Song L., Liang J., Cao N., Gao J., Xu R., Ma Y. (2021). Curcumin Reverses Hepatic Epithelial Mesenchymal Transition Induced by Trichloroethylene by Inhibiting IL-6R/STAT3. Toxicol. Mech. Methods.

[251] Kunnumakkara A.B., Diagaradjane P., Anand P., Kuzhuvelil H.B., Deorukhkar A., Gelovani J., Guha S., Krishnan S., Aggarwal B.B. (2009). Curcumin Sensitizes Human Colorectal Cancer to Capecitabine by Modulation of Cyclin D1, COX-2, MMP-9, VEGF and CXCR4 Expression in an Orthotopic Mouse Model. Int. J. Cancer.

[252] Goel A., Kunnumakkara A.B., Aggarwal B.B. (2008). Curcumin as “Curecumin”: From Kitchen to Clinic. Biochem. Pharmacol..

[253] Shao M., Lou D., Yang J., Lin M., Deng X., Fan Q. (2020). Curcumin and Wikstroflavone B, a New Biflavonoid Isolated from Wikstroemia Indica, Synergistically Suppress the Proliferation and Metastasis of Nasopharyngeal Carcinoma Cells via Blocking FAK/STAT3 Signaling Pathway. Phytomedicine.

[254] Xu T., Guo P., Pi C., He Y., Yang H., Hou Y., Feng X., Jiang Q., Wei Y., Zhao L. (2020). Synergistic Effects of Curcumin and 5-Fluorouracil on the Hepatocellular Carcinoma In Vivo and Vitro through Regulating the Expression of COX-2 and NF-ΚB. J. Cancer.

[255] Howells L.M., Iwuji C.O.O., Irving G.R.B., Barber S., Walter H., Sidat Z., Griffin-Teall N., Singh R., Foreman N., Patel S.R. (2019). Curcumin Combined with FOLFOX Chemotherapy Is Safe and Tolerable in Patients with Metastatic Colorectal Cancer in a Randomized Phase IIa Trial. J. Nutr..

[256] Tian B., Wang Z., Zhao Y., Wang D., Li Y., Ma L., Li X., Li J., Xiao N., Tian J. (2008). Effects of Curcumin on Bladder Cancer Cells and Development of Urothelial Tumors in a Rat Bladder Carcinogenesis Model. Cancer Lett..

[257] Hartojo W., Silvers A.L., Thomas D.G., Seder C.W., Lin L., Rao H., Wang Z., Greenson J.K., Giordano T.J., Orringer M.B. (2010). Curcumin Promotes Apoptosis, Increases Chemosensitivity, and Inhibits Nuclear Factor KappaB in Esophageal Adenocarcinoma. Transl. Oncol..

[258] Mohammadian F., Pilehvar-Soltanahmadi Y., Mofarrah M., Dastani-Habashi M., Zarghami N. (2016). Down Regulation of MiR-18a, MiR-21 and MiR-221 Genes in Gastric Cancer Cell Line by Chrysin-Loaded PLGA-PEG Nanoparticles. Artif. Cells Nanomed. Biotechnol..

[259] Vinod B.S., Antony J., Nair H.H., Puliyappadamba V.T., Saikia M., Shyam Narayanan S., Bevin A., John Anto R. (2013). Mechanistic Evaluation of the Signaling Events Regulating Curcumin-Mediated Chemosensitization of Breast Cancer Cells to 5-Fluorouracil. Cell Death Dis..

[260] Kim C.H., Kim B.D., Lee T.H., Kim H.K., Lyu M.J., Yoon Y.I., Goo Y.T., Kang M.J., Lee S., Choi Y.W. (2022). Synergistic Co-Administration of Docetaxel and Curcumin to Chemoresistant Cancer Cells Using PEGylated and RIPL Peptide-Conjugated Nanostructured Lipid Carriers. Cancer Nanotechnol..

[261] Zoi V., Galani V., Lianos G.D., Voulgaris S., Kyritsis A.P., Alexiou G.A. (2021). The Role of Curcumin in Cancer Treatment. Biomedicines.

[262] Kunnumakkara A.B., Anand P., Aggarwal B.B. (2008). Curcumin Inhibits Proliferation, Invasion, Angiogenesis and Metastasis of Different Cancers through Interaction with Multiple Cell Signaling Proteins. Cancer Lett..

[263] Yoshida K., Toden S., Ravindranathan P., Han H., Goel A. (2017). Curcumin Sensitizes Pancreatic Cancer Cells to Gemcitabine by Attenuating PRC2 Subunit EZH2, and the LncRNA PVT1 Expression. Carcinogenesis.

[264] Sandhiutami N.M.D., Arozal W., Louisa M., Rahmat D., Wuyung P.E. (2021). Curcumin Nanoparticle Enhances the Anticancer Effect of Cisplatin by Inhibiting PI3K/AKT and JAK/STAT3 Pathway in Rat Ovarian Carcinoma Induced by DMBA. Front. Pharmacol..

[265] Ghiringhelli F., Chibaudel B., Taieb J., Bennouna J., Martin-Babau J., Fonck M., Borg C., Cohen R., Thibaudin M., Limagne E. (2020). Durvalumab and Tremelimumab in Combination with FOLFOX in Patients with RAS-Mutated, Microsatellite-Stable, Previously Untreated Metastatic Colorectal Cancer (MCRC): Results of the First Intermediate Analysis of the Phase Ib/II MEDETREME Trial. J. Clin. Oncol..

[266] Batra H., Pawar S., Bahl D. (2019). Curcumin in Combination with Anti-Cancer Drugs: A Nanomedicine Review. Pharmacol. Res..

[267] Kabir M.T., Rahman M.H., Akter R., Behl T., Kaushik D., Mittal V., Pandey P., Akhtar M.F., Saleem A., Albadrani G.M. (2021). Potential Role of Curcumin and Its Nanoformulations to Treat Various Types of Cancers. Biomolecules.

[268] Sohn S.I., Priya A., Balasubramaniam B., Muthuramalingam P., Sivasankar C., Selvaraj A., Valliammai A., Jothi R., Pandian S. (2021). Biomedical Applications and Bioavailability of Curcumin—An Updated Overview. Pharmaceutics.

[269] Joe B., Vijaykumar M., Lokesh B.R. (2004). Biological Properties of Curcumin-Cellular and Molecular Mechanisms of Action. Crit. Rev. Food Sci. Nutr..

[270] Hewlings S.J., Kalman D.S. (2017). Curcumin: A Review of Its Effects on Human Health. Foods.

[271] Ojo O.A., Adeyemo T.R., Rotimi D., Batiha G.E.S., Mostafa-Hedeab G., Iyobhebhe M.E., Elebiyo T.C., Atunwa B., Ojo A.B., Lima C.M.G. (2022). Anticancer Properties of Curcumin Against Colorectal Cancer: A Review. Front. Oncol..

[272] Imran M., Saeed F., Alsagaby S.A., Imran A., Ahmad I., El Ghorab A.H., Abdelgawad M.A., Qaisrani T.B., Mehmood T., Umar M. (2023). Curcumin: Recent Updates on Gastrointestinal Cancers. CYTA J. Food.

[273] Song X., Zhang M., Dai E., Luo Y. (2019). Molecular Targets of Curcumin in Breast Cancer (Review). Mol. Med. Rep..

[274] Schmidt K.T., Figg W.D. (2016). The Potential Role of Curcumin in Prostate Cancer: The Importance of Optimizing Pharmacokinetics in Clinical Studies. Transl. Cancer Res..

[275] Hu A., Huang J.J., Li R.L., Lu Z.Y., Duan J.L., Xu W.H., Chen X.P., Fan J.P. (2015). Curcumin as Therapeutics for the Treatment of Head and Neck Squamous Cell Carcinoma by Activating SIRT1. Sci. Rep..

[276] Omidian H., Wilson R.L., Chowdhury S.D. (2023). Enhancing Therapeutic Efficacy of Curcumin: Advances in Delivery Systems and Clinical Applications. Gels.

[277] Ahn J.C., Kang J.W., Shin J.I., Chung P.S. (2012). Combination Treatment with Photodynamic Therapy and Curcumin Induces Mitochondria-Dependent Apoptosis in AMC-HN3 Cells. Int. J. Oncol..

[278] Dujic J., Kippenberger S., Hoffmann S., Ramirez-Bosca A., Miquel J., Diaz-Alperi J., Bereiter-Hahn J., Kaufmann R., Bernd A. (2007). Low Concentrations of Curcumin Induce Growth Arrest and Apoptosis in Skin Keratinocytes Only in Combination with UVA or Visible Light. J. Investig. Dermatol..

